# Identification of TENM4 as a Novel Cancer Stem Cell-Associated Molecule and Potential Target in Triple Negative Breast Cancer

**DOI:** 10.3390/cancers13040894

**Published:** 2021-02-20

**Authors:** Roberto Ruiu, Giuseppina Barutello, Maddalena Arigoni, Federica Riccardo, Laura Conti, Giulia Peppino, Laura Annaratone, Caterina Marchiò, Giulio Mengozzi, Raffaele Adolfo Calogero, Federica Cavallo, Elena Quaglino

**Affiliations:** 1 Molecular Biotechnology Center, Department of Molecular Biotechnology and Health Sciences, University of Torino, Via Nizza 52, 10126 Torino, Italy; roberto.ruiu@unito.it (R.R.); giuseppina.barutello@unito.it (G.B.); maddalena.arigoni@unito.it (M.A.); federica.riccardo@unito.it (F.R.); laura.conti@unito.it (L.C.); giulia.peppino@unito.it (G.P.); raffaele.calogero@unito.it (R.A.C.); federica.cavallo@unito.it (F.C.); 2 Unit of Pathology, Candiolo Cancer Institute, FPO IRCCS, 10060 Candiolo, Italy; laura.annaratone@unito.it (L.A.); caterina.marchio@unito.it (C.M.); 3 Department of Medical Sciences, University of Torino, 10126 Torino, Italy; giulio.mengozzi@unito.it; 4 Clinical Biochemistry Laboratory, Department of Laboratory Medicine, AOU Città della Salute e della Scienza di Torino, 10126 Torino, Italy

**Keywords:** cancer stem cell (CSC), triple negative breast cancer (TNBC), transcriptomics, teneurin 4 (TENM4)

## Abstract

**Simple Summary:**

Patients with triple negative breast cancer (TNBC) experience shorter overall survival compared to non-TNBC patients because of the high incidence of recurrences and metastases. This is due to the capacity of aggressive cancer cell subpopulations named cancer stem cells (CSC) to resist current therapies. To design more effective therapeutic strategies for TNBC patients, in this study we sought to identify functional targets expressed on CSC. Our analyses led us to propose teneurin 4 (TENM4) as a promising candidate for drug- and immune-based therapies due to its role in CSC self-renewal and migratory capacity and the inverse correlation between its expression and survival of TNBC patients. In addition, TENM4 detection in the plasma of tumor-bearing patients endorses its potentiality as a disease detection marker.

**Abstract:**

Triple-negative breast cancer (TNBC) is insensitive to endocrine and Her2-directed therapies, making the development of TNBC-targeted therapies an unmet medical need. Since patients with TNBC frequently show a quicker relapse and metastatic progression compared to other breast cancer subtypes, we hypothesized that cancer stem cells (CSC) could have a role in TNBC. To identify putative TNBC CSC-associated targets, we compared the gene expression profiles of CSC-enriched tumorspheres and their parental cells grown as monolayer. Among the up-regulated genes coding for cell membrane-associated proteins, we selected Teneurin 4 (TENM4), involved in cell differentiation and deregulated in tumors of different histotypes, as the object for this study. Meta-analysis of breast cancer datasets shows that TENM4 mRNA is up-regulated in invasive carcinoma specimens compared to normal breast and that high expression of TENM4 correlates with a shorter relapse-free survival in TNBC patients. TENM4 silencing in mammary cancer cells significantly impaired tumorsphere-forming ability, migratory capacity and Focal Adhesion Kinase (FAK) phosphorylation. Moreover, we found higher levels of TENM4 in plasma from tumor-bearing mice and TNBC patients compared to the healthy controls. Overall, our results indicate that TENM4 may act as a novel biomarker and target for the treatment of TNBC.

## 1. Introduction

Triple negative breast cancer (TNBC) is the most aggressive type of human breast cancer and affects 15–20% of breast cancer patients [1]. It is characterized by the lack of expression of estrogen receptor (ER), progesterone receptor (PR) and epidermal growth factor receptor (Her2). Compared with other subgroups of breast cancer patients, those with TNBC have a significantly higher risk of recurrence, distant metastases and death within 5 years from diagnosis [2]. Being negative for the expression of ER, PR and Her2, TNBC is insensitive to both endocrine and Her2-directed therapies. The current lack of routine targeted therapies for TNBC leaves non-selective cytotoxic chemotherapy the main stay of treatment. However, despite having a higher response rate to chemotherapy compared to non-TNBC, TNBC patients with residual disease, which represent 60–70% of chemotherapy-treated patients [3], usually have a shorter overall survival driven by higher relapse rate compared to non-TNBC-patients, a phenomenon described as “TNBC paradox” [4].

Nowadays, the tracing of TNBC biomarkers represents a potent tool to guide towards targeted therapies and immunotherapeutic approaches [5]. Indeed, different TNBC subtypes have shown to be sensitive to distinct treatment approaches. For example, TNBC patients harboring inactivating mutations in BRCA1 or BRCA2 genes have shown to benefit from platinum-based chemotherapy [6,7] and from using PARP inhibitors [8,9,10]. Moreover, mutations in the phosphoinositide 3-kinase (PI3K) pathway, found in about 25% of primary TNBC and at a modestly higher frequency in metastatic TNBC, suggest the possible efficacy of targeted interventions against PI3K and its downstream interactors [11]. Data from randomized trials suggest improved progression-free survival when AKT inhibitors in combination with first-line chemotherapy were administered in patients with TNBC harboring PI3K pathway genetic aberrations. However, robust evidence of efficacy is still lacking, since for many of these studies results are not yet available and therefore conclusions cannot be drawn [11]. Recently, also immune biomarkers have been considered as proper targets in TNBC; indeed, the combined use of a monoclonal antibody directed against programmed cell death ligand 1 (PDL1), atezolizumab, and nab-paclitaxel, has demonstrated its efficacy in patients affected by unresectable locally advanced TNBC infiltrated with PDL1-positive immune cells [12]. Standing out the complexity of TNBC, it is clear that uncovering new specific TNBC biomarkers is needed to stratify TNBC patients and replace unselective chemotherapy treatment.

In recent years, cancer stem cells (CSC) have gained intense interest as they are thought to play a pivotal role in recurrence following chemotherapy [13]. CSC are a small population of tumor-initiating cells that are endowed with the ability to both self-renew and differentiate into non-tumorigenic daughter cells that constitute the bulk of the tumor. Conventional treatments—i.e., chemo- and radiotherapy—designed to shrink the bulk of a tumor may fail to eliminate the small fraction of CSC, or even drive the evolution of differentiated tumor cells towards the CSC phenotype [14]. Therefore, the final success of a treatment may rest on complete CSC suppression [15] and the identification of appropriate molecular targets expressed by CSC represents the critical point in the development of more effective anticancer therapies.

Taking advantage of our workflow developed for the identification of transcripts expressed by Her2+ mammary CSC [16,17,18,19] in the present study we performed a transcriptome analysis of cancer cells grown as a monolayer (hereafter named epithelial cells) and CSC-enriched tumorspheres derived from mouse and human TNBC cell lines. We focused our attention on proteins overexpressed on the cell membrane of TNBC stem cells since this kind of protein can be used as targets for both drug- and immune-based anti-cancer therapies. This analysis led to the identification of differentially regulated biological processes between epithelial cells and tumorspheres, as well as of a novel candidate TNBC-associated target, a large transmembrane protein belonging to the teneurin protein family, named Teneurin 4 (TENM4). TENM4 expression increases from epithelial cells to tumorspheres, providing them advantages in self-renewal and migration ability. Beside its well described role in organogenesis [20] and in the development of the nervous system [21,22], TENM4 has been recently liked with cancer where its deregulated expression correlates with patients’ prognosis [23,24]. Moreover, the protein family of teneurins seems to have a role in tumorigenesis thanks to the ability of its members, including TENM4, to modulate cell differentiation [23,25].

Considering the key role played by CSC in cancer progression and resistance to current therapies, as well as the importance of their targeting through surface antigens, our discovery of TENM4 as a new TNBC stem cell marker offers new opportunities for the development of effective anti-cancer therapy.

## 2. Results

### 2.1. Tumorspheres Generation and Characterization

**Figure 1:**
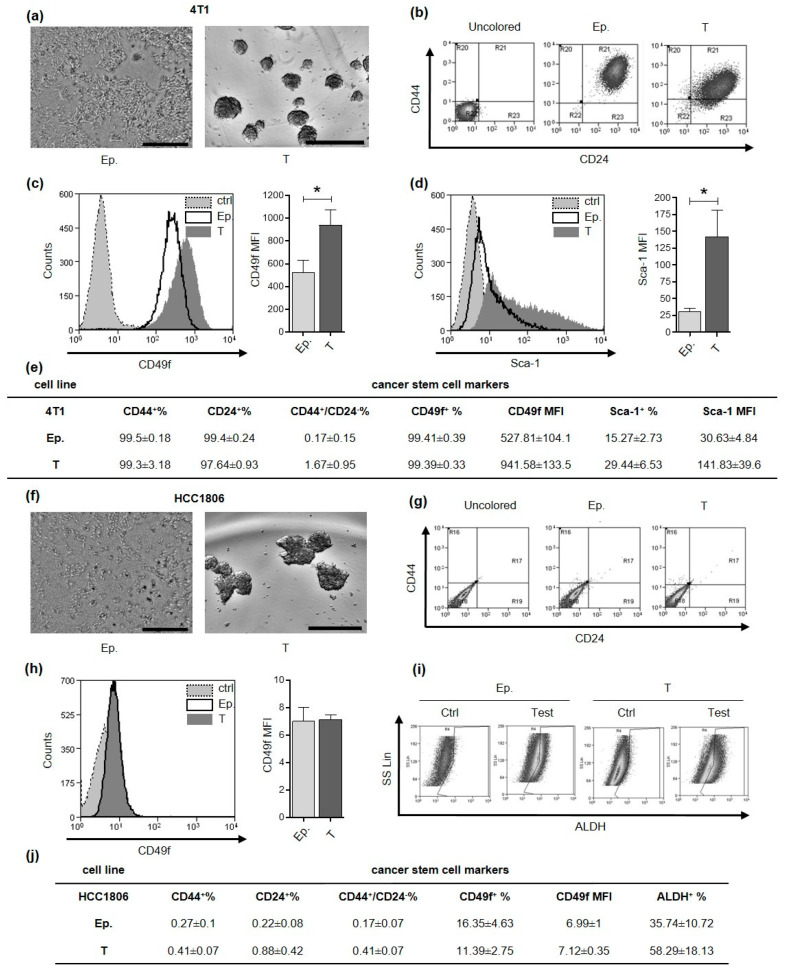
Tumorspheres generation and characterization. CSC markers characterization of 4T1 and HCC1806 cell lines. (**a**,**f**) Optical microscope pictures of sub-confluent epithelial cells and 5-day-old tumorspheres are reported. Grey scale bar: 500 µm. (**b**,**g**) density plots represent the flow cytometry double staining of epithelial (Ep.) or tumorsphere-derived (T) cells for CD44 and CD24 surface markers. Flow cytometry histogram representations for CD49f (**c**,**h**) and Sca-1 (**d**). Gray fills with dotted lines refer to unstained control cells, no fills with black lines to Ep. cells and dark gray fills with no lines to T cells. The cell count is reported on the y-axis, the fluorescence intensity of the staining for the indicated surface marker is reported on the x-axis. Bar graphs show comparison of Mean Fluorescence Intensity (MFI) for a given marker between Ep. and T cells. (**e**,**j**) Tables represent the mean percentage of positive Ep. or T cells for a given marker and the MFI of a given marker ± SEM, as assessed by flow cytometry. (**i**) Density plot representation of HCC1806 cell staining for Aldehyde Dehydrogenase (ALDH) activity reports fluorescence on x-axis and side scatter (SS Lin) on the y-axis. * *p* < 0.05.

4T1 and HCC1806 cell lines were selected as model for murine and human TNBC, respectively. Based on culture conditions previously established for these and other breast cancer cell lines [17,18,19], we successfully generated CSC-enriched tumorspheres (Figure 1a,f). Breast CSC can be defined and isolated through the evaluation of different mammary stem cell marker expression among which CD44+/CD24−, Sca-1+, CD49f+ and Aldehyde Dehydrogenase (ALDH) activity. When we analyzed these markers in 4T1 epithelial and tumorsphere-derived cells by flow cytometry we found that almost 100% of the cells were positive for both CD44 and CD24 expression (Figure 1b,e). Conversely, the majority of HCC1806 cells grown as monolayer or tumorspheres, displayed negativity either for CD44 or CD24 (Figure 1g,j). Nevertheless, tumorsphere-derived cells from HCC1806 have a trend of increase in ALDH activity compared to cells grown as monolayers (Figure 1i,j), while 4T1 tumorsphere-derived cells displayed a significantly higher mean fluorescence intensity (MFI) for CD49f and Sca-1 as compared to epithelial cells (Figure 1c–e). By contrast, CD49f was expressed to a similar extent in both epithelial and tumorsphere-derived cells in HCC1806 (Figure 1h,j). These data indicate that markers commonly used for the characterization of breast CSC display a high degree of heterogeneity in the two cell lines tested, with CD49f and Sca-1 being more appropriate for 4T1 CSC and ALDH for HCC-1806 CSC.

### 2.2. Identification of Down-Regulated and Up-Regulated Gene Sets in TNBC Tumorsphere-Derived Cells

**Figure 2:**
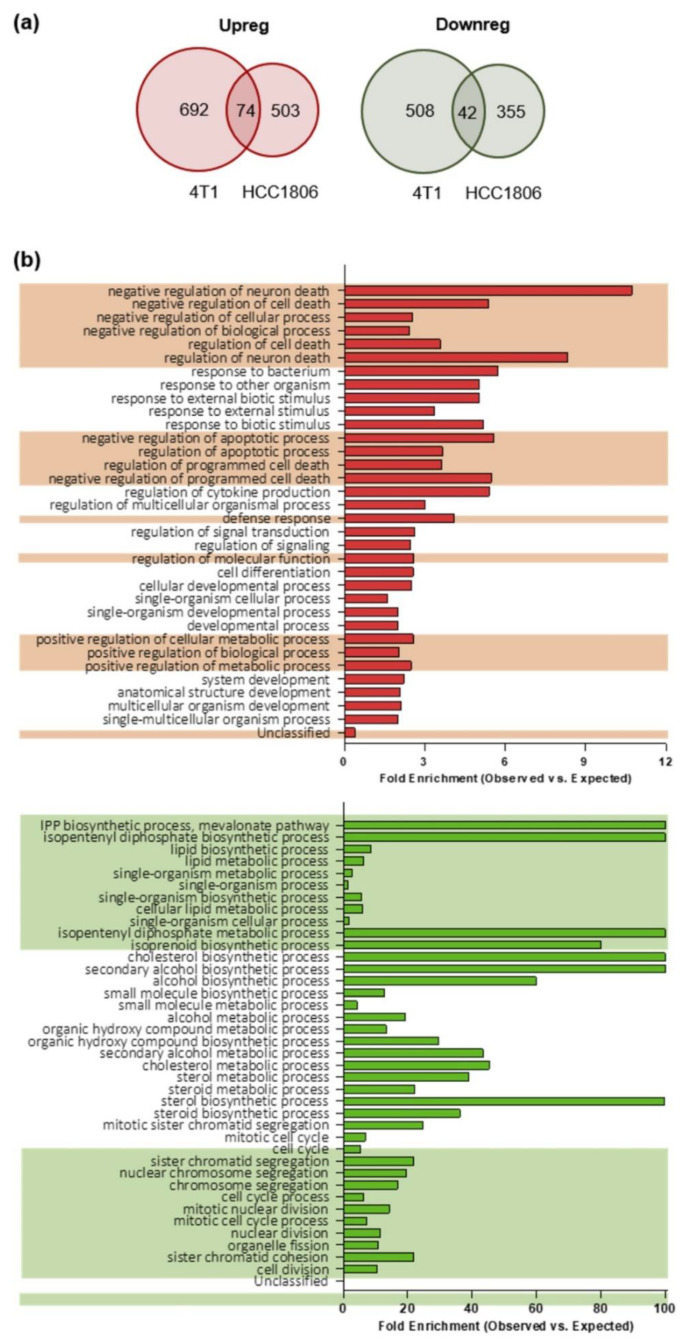
Gene expression profiling and gene ontology (GO) biological processes of epithelial and tumorsphere-derived cells. (**a**) Venn-diagrams representing the number of up-regulated (Upreg; red) or down-regulated (Downreg; green) genes shared between 4T1 and HCC1806 cell lines. (**b**) Histograms representing the distribution of the genes according to their biological function. The reported classes are GO biological processes. In red the analysis of the 4T1 and HCC1806 commonly up-regulated transcripts while in green that of the down-regulated ones. The bars represent the ratio between the number of genes observed for a given biological process (Observed) versus the number of genes that would be observed by chance (Expected) for that same biological process. The alternating background helps to visualize biological processes that are related, and that can be interpretable as a group rather than individually (obtained by the hierarchic sort function of GO).

To compare the gene expression profiles of TNBC stem cells-enriched tumorspheres and their epithelial counterpart, we applied a modified pipeline previously developed by us to compare the transcriptome of breast CSC-enriched tumorspheres with that of their more differentiated counterparts [16] in order to identify TNBC CSC-associated antigens. RNA from epithelial and tumorsphere-derived cells was extracted and sequenced, and RNA sequencing data were analyzed as reported in the “material and methods” section. We considered as differentially expressed only genes whose log_2_ fold change in expression was either ≤−1 (down-regulated in tumorspheres) or ≥1 (up-regulated in tumorspheres) with an adjusted p-value ≤0.1. Similar percentages of differentially expressed transcripts were found between epithelial and tumorsphere-derived 4T1 (13.6%) and HCC1806 (8.6%) cells. Moreover, for each cell line also the proportions of up-regulated (1.7% for 4T1 and 1.5% for HCC1806 cells) and down-regulated (2.3% for 4T1 and 2.9% for HCC1806 cells) transcripts among the differentially expressed ones were not significantly different. To study the role of potential CSC-associated transcripts in preclinical models of TNBC and to evaluate in further studies the impact of their immune targeting in vivo, we narrowed the field of analysis only to the minor proportion of differentially expressed transcripts shared between 4T1 and HCC1806 cell lines. In summary, 74 transcripts were up-regulated in the tumorspheres of both 4T1 and HCC1806 cells, while 42 transcripts were found down-regulated (Figure 2a).

To better understand the biological implications of the molecular events characterizing the enrichment of CSC within tumorspheres, the two sets of genes detected as differentially expressed between epithelial cell lines and the corresponding tumorspheres were analyzed through the Gene Ontology (GO) enrichment analysis tool. For a complete list of biological functions enriched in the gene sets, and their related genes belonging to the gene sets, see Tables S1 and S2. An enrichment in genes involved in the negative regulation of apoptosis was found among the up-regulated gene set in tumorspheres as compared to epithelial cells (Figure 2b, upper panel). It is worth noting that many of the genes in the up-regulated set encode proteins with pleiotropic functions, which are thus involved in different biological processes. For instance, NR4A2, which is up-regulated in tumorspheres (Table S1), is not only related to negative regulation of apoptosis, but also to positive regulation of biosynthetic process, neurogenesis, response to stress, cell communication and many others. Within the gene set down-regulated in tumorspheres, an enrichment in genes involved in lipid and cholesterol biosynthesis and cell cycle regulation (Figure 2b, lower panel) was observed. 

Taken together, our data suggest that CSC are more resistant to apoptosis compared to epithelial cells, since genes that are involved in the negative regulation of apoptosis are up-regulated in tumorspheres as compared to epithelial cells. Moreover, since key genes required for the progression of the cell cycle through the G2/M phase and for the biosynthesis of cholesterol and of lipids (Figure 2b) are significantly down-regulated in tumorspheres, our data demonstrate that CSC-enriched tumorspheres are in a relatively quiescent state accompanied by a reduced biosynthesis of cholesterol and lipids.

### 2.3. TENM4 Emerges as a Novel Breast CSC-Associated Molecule

When looking for candidate targets among the up-regulated genes, we narrowed our search only to genes whose protein products are predicted to be expressed on the cell surface. This approach was chosen since transmembrane proteins represent the ideal targets also for antibody-based therapies [14]. Among the 74 up-regulated transcripts, only 17 genes encoded for transmembrane proteins (Table 1).

**Figure 3:**
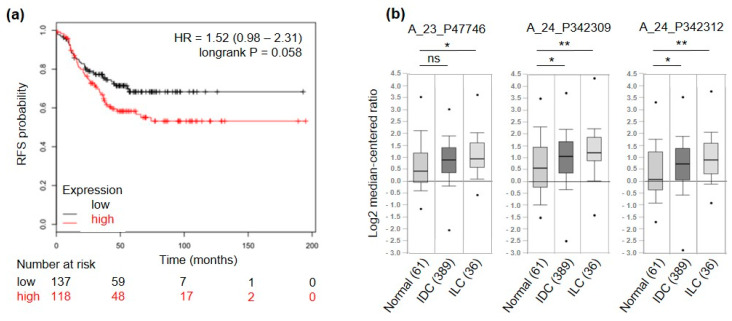
TENM4 relevance in breast cancer patients. (**a**) RFS of TNBC patients, stratified by high (red) or low (black) expression of TENM4 mRNA. (**b**) Oncomine TENM4 mRNA expression data reported as log2 median-centered ratio using box-and-whiskers plots (dots: maximum and minimum values; whiskers: 90/10 percentiles; box: 75/25 percentiles; line: median of all samples). Expression data from normal breast tissue (61 samples) were compared with those from invasive ductal carcinoma (IDC, 389 samples) and invasive lobular carcinoma (ILC, 36 samples). The analysis was performed for three different Agilent ID probes. * *p* < 0.05; ** *p* < 0.01; ns: non-significant.

To further select only genes of interest for TNBC patients, a meta-analysis on a set of publicly available microarray data from breast cancer patients [26] was run. High expression levels of 5 out of the 17 candidate genes were related to good prognosis in terms of relapse-free survival (RFS) in TNBC patients (3 significantly and 2 non-significantly) and were therefore excluded. Among the remaining 12 candidates, 3 were significantly associated (ACVR2B, COMTD1 and F11R, *p*-value < 0.05) and 6 displayed a trend of association (IGF1R, ITGA10, PIP5K1C, PMP, SSC5D, TENM4, *p*-value ≤ 0.1) to shorter RFS (Figure 3a and Figure S1). Among these 12 candidate genes we found TENM4 of particular interest. Indeed, TENM4 resulted one of the top ranking differentially expressed genes between CSC-enriched tumorspheres and epithelial murine and human TNBC cells (>7-fold increase in 4T1 and >5-fold increase in HCC1806; Table 1). In addition, by querying for TENM4 expression in the TCGA database through Oncomine (see “material and methods” section), TENM4 mRNA resulted to be expressed at higher levels in both ductal and lobular invasive carcinoma of the breast compared to normal breast (Figure 3b). Considering that a high percentage of both ductal and lobular invasive carcinomas of the breast are estrogen receptor positive, TENM4 could be a relevant target not only in TNBC but also in other breast cancer subtypes.

Given these premises, we decided to further investigate the role of TENM4 in murine and human TNBC models, persuaded that TENM4 fulfills the characteristics of a good candidate as an unexplored CSC-associated molecule.

### 2.4. TENM4 Expression Increases in Murine and Human TNBC Tumorspheres

**Figure 4:**
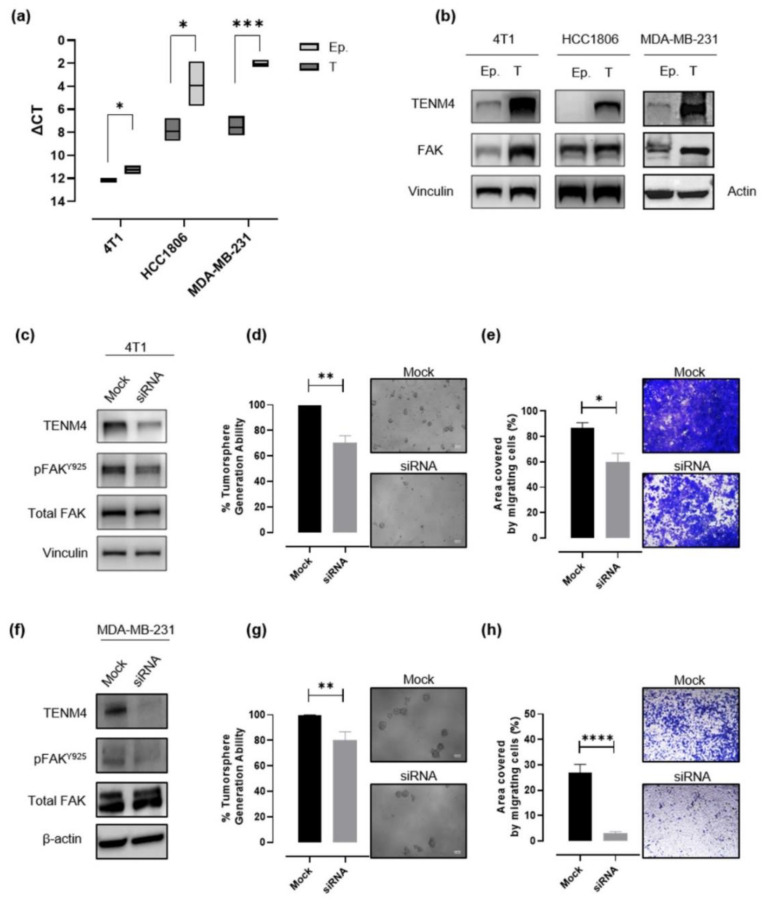
Evaluation of the role of TENM4 in tumorspheres. (**a**) Semi-quantitative RT-PCR. Boxes represent the ΔCt (i.e., the difference between the Ct of TENM4 and the Ct of the internal control gene GADPH), comparing epithelial (Ep.) and tumorsphere-derived (T) cells. The inner line of the box corresponds to the mean value, upper and lower edges correspond to higher and lower value, respectively. (**b**) Immunoblot of TENM4, total FAK and loading control protein, vinculin or β-actin, comparing 4T1, HCC1806 and MDA-MB-231 epithelial (Ep.) and tumorsphere-derived (T) cells. (**c**,**f**) Immunoblot of TENM4, total FAK, FAK phosphorylated at Y925 and loading control protein, vinculin or β-actin, comparing 4T1 or MDA-MB-231 epithelial cells, respectively, treated with a pool of TENM4-specific siRNA (siRNA) or a pool of non-targeting siRNA (mock). (**d**,**g**) Histograms showing the sphere-generation ability and representative pictures of the formed tumorspheres after TENM4 silencing in 4T1 and MDA-MB-231 cells, respectively. The mean values in the mock siRNA-treated condition were used as reference and considered to be 100%. Values of TENM4 silenced cells represent the % of the tumorsphere forming ability respect the mean value of the mock siRNA-treated cells. White scale bar: 100 µm. (**e**,**h**) Effect of TENM4 silencing on 4T1 and MDA-MB-231 cell migratory ability, assessed by using the Transwell migration assay. Histograms showing the mean ± SEM of the percentage of the area covered by migrating siRNA and mock cells and representative pictures of crystal violet-stained migrating cells are reported for 4T1 and MDA-MB-231 cells, respectively. Statistical analysis was carried out with Student’s *t* test: * *p* < 0.05; ** *p* ≤ 0.006; *** *p* < 0.001; **** *p* < 0.0001.

In order to validate data obtained through RNA sequencing, TENM4 mRNA levels were evaluated by a semi quantitative real time (RT)-PCR in epithelial and tumorsphere-derived cells from 4T1, HCC1806 and from another human TNBC cell line, the MDA-MB-231, from which we successfully obtained CSC-enriched tumorspheres, as demonstrated by their increased ALDH activity as compared to the epithelial counterpart (Figure S2a,b). A significant TENM4 mRNA up-regulation was found in all the TNBC tumorsphere-derived cells tested as compared to their epithelial counterpart (Figure 4a), confirming data obtained from RNA sequencing. Accordingly, TENM4 up-regulation was validated at the protein level in both mouse 4T1 and human HCC1806 and MDA-MB-231 cells (Figure 4b). Interestingly, TENM4 mRNA up-regulation was not restricted only to TNBC tumorsphere-derived cells but was also evident in tumorspheres from MCF-7 (human ER+) and SK-BR-3 (human Her2+) breast cancer cell line (Figure S3a), confirming a potential role of TENM4 not only in TNBC but also in other breast cancer subtypes.

### 2.5. TENM4 Up-Regulation May Be Functionally Related to CSC Self-Renewal and to the Migratory Capacity of TNBC through Focal Adhesion Kinase

Since TENM4 has proven to be functionally related to Focal Adhesion Kinase (FAK) both in non-transformed [21,27] and in cancer cells [28], and increased expression of FAK has been reported to be an important feature of cells with a stem-like phenotype [29], we investigated FAK expression in TNBC epithelial cells and tumorspheres. Although, in the case of HCC1806 cells we were not able to reveal a substantive difference in FAK expression levels between epithelial and tumorsphere-derived cells (Figure 4b; middle panel), a significant increase in FAK expression in tumorspheres compared to epithelial cells was observed in 4T1 and MDA-MB-231 cells (Figure 4b).

FAK is known to be involved in cell adhesion and motility, being the major downstream molecule of integrin signaling following interaction between cells and the extracellular matrix (ECM), and TENM4 has been shown to interact with component of the ECM suppressing cell differentiation and preserving a mesenchymal phenotype [27]. For these reasons we investigated if TENM4 expression could affect CSC self-renewal and cell migration, taking advantage of RNA interference technology.

As expected, the treatment with a pool of TENM4 specific siRNA resulted in a significant decrease in TENM4 mRNA and protein levels in 4T1 (Figure 4c), MDA-MB-231 (Figure 4f) and HCC1806 (Figure S4a) cells. In TENM4-silenced 4T1 and MDA-MB-231 cells we also evaluated the phosphorylation of FAK at tyrosine 925 site (Figure 4c,f), demonstrating its decrease as compared to unspecific siRNA-treated cells. In addition, TENM4 silencing led to a partial but significant impairment of 4T1 (Figure 4d), MDA-MB-231 (Figure 4g) and HCC1806 (Figure S4b) tumorsphere-forming ability. Interestingly, a reduction of stemness marker expression (Figure S5) was also observed. In particular, TENM4 silencing in 4T1 tumorsphere-derived cells resulted in a decrease in the percentage of CD44^+^CD24^-^ cells (Figure S5a), a reduction of the expression of the octamer-binding transcription factor 4 (OCT4) (Figure S5b), responsible for CSC self-renewal and pluripotency [30] and a down-regulation of CD49f expression (Figure S5c). Similarly, when TENM4 was silenced in human MDA-MB-231 (Figure S5d) and HC1806 (Figure S5e,f) cells a lower ALDH activity and/or OCT4 expression was observed as compared to unspecific siRNA treated cells. Finally, when 4T1 (Figure 4e), MDA-MD-231 (Figure 4h) and HCC1806 (Figure S4c) cells treated with TENM4 specific siRNA were tested in a migration assay in vitro, a significantly reduced ability to migrate was demonstrated.

Taken together these results suggest a role of TENM4 in the maintenance of self-renewal and a possible link between TENM4 and a CSC-like phenotype in TNBC.

### 2.6. TENM4 is Detectable in The Plasma of TNBC Tumor-Bearing Mice and Patients

**Figure 5:**
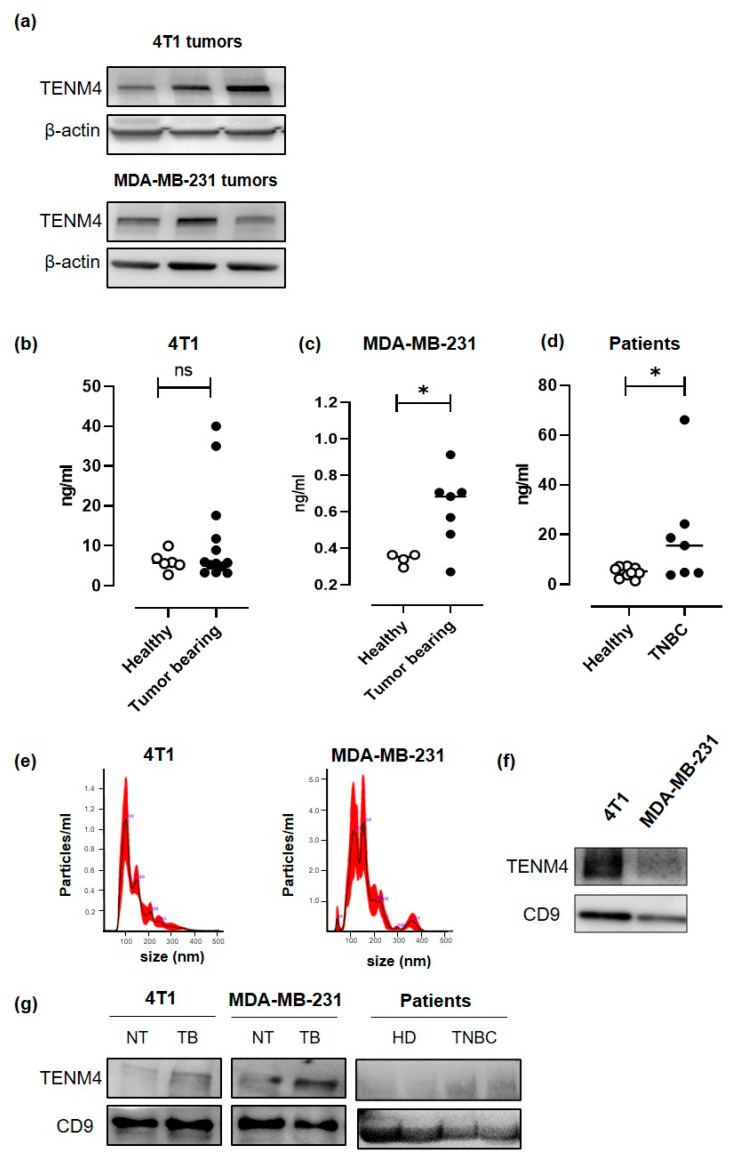
Evaluation of the role of TENM4 as TNBC biomarker. (**a**) Representative immunoblot of TENM4 and loading control protein, β-actin, in tumors derived from the subcutaneous injection of 4T1 (upper panel) or MDA-MB-231 (lower panel) cells. (**b**) ELISA assay revealing the presence of TENM4 protein in the plasma of tumor-bearing mice injected with 4T1 (left panel; *n* = 13), (**c**) MDA-MB-231 (central panel; *n* = 7) cells or (**d**) in TNBC patients (right panel; *n* = 7) comparing to healthy subjects (Healthy; *n* = 13; *n* = 7; *n* = 10, respectively). (**e**) Representative graphs of nanoparticle tracking analysis and (**f**) representative immunoblot of TENM4 and CD9 of extracellular vesicles (EVs) collected from 4T1 and MDA-MB-231 conditioned media. (**g**) Representative immunoblot of TENM4, CD9 and CD63 assessing their expression in plasma-derived EVs collected from 4T1-, MDA-MB-231-derived tumor-bearing (TB) or not treated (NT) mice or from TNBC patients compared to healthy donors (HD). Statistical analysis was carried out with Student’s *t*-test: * *p* < 0.05.

Since a proteomic analysis demonstrated the presence of soluble forms of TENM4 in the human urine [31], we checked for circulating TENM4 in the plasma of TENM4^+^ TNBC tumor-bearing mice. 4T1 and MDA-MB-231 cells were injected subcutaneously in BALB/c and in NOD/SCID-gamma null (NSG) mice, respectively, and TENM4 protein expression was tested by Western blot in the lysates from 8–10 mm mean diameter tumors. Heterogeneous level of TENM4 protein was detected in all the established 4T1 and MDA-MB-231 tumors (Figure 5a), demonstrating that TENM4 protein expression is not restricted to in vitro cultured cell lines (Figure 4b). To test whether TENM4 shedding and/or release occurs in vivo, we checked TENM4 protein in the plasma from TNBC bearing mice. While a trend of increase in circulating TENM4 was observed in 4T1 tumor-bearing as compared to healthy BALB/c mice (Figure 5b), significantly higher amounts of TENM4 were found in the plasma of MDA-MB-231 tumor-bearing mice as compared to healthy NSG mice (Figure 5c). Preliminary results from the small cohort of 48 breast cancer patients (Figure S3b), including 7 TNBC patients (Figure 5d), showed significantly higher amount of TENM4 in the plasma of tumor-bearing patients as compared to healthy donors.

TENM4 can also be present in the tumor-derived exosomes, as demonstrated in neuroblastoma cancer cells [32]. Thus, we explored the possibility of TENM4 vehiculation through extracellular vesicles (EVs) from 4T1 and MDA-MB-231 TNBC cell lines. Purified EVs (89–235 nm and 91–260 nm for 4T1 and MDA-MB231, respectively; Figure 5e) were lysed and tested by Western blot, demonstrating TENM4 expression in EVs from both 4T1 and MDA-MB-231 cells. CD9, a member of the tetraspanin family was used as EVs biomarker (Figure 5f). We then focused on plasma-derived EVs from 4T1 and MDA-MB-231 tumor-bearing mice, demonstrating higher amounts of TENM4 as compared to plasma-derived EVs from healthy mice (Figure 5g). Finally, similar results were obtained when we analyzed plasma-derived EVs from TNBC patients (Figure 5g) of our breast cancer patients’ cohort (Figure S3c). CD9 and CD63 were used as EVs and exosomes biomarker, respectively (Figure 5g).

Taken together these findings suggest that circulating TENM4 could be a potential disease biomarker for breast cancer that deserves further investigation.

## 3. Discussion

The purpose of this study was to identify candidate targets—for drug- and immune-based therapies—expressed by breast CSC. Indeed, CSC elimination or their permanent functional suppression is required for the success of anti-tumor treatments, being CSC considered the source of primary tumors and metastases [33]. To this aim we took advantage of the pipeline we previously developed and used for the identification of murine Her2+ breast CSC antigens [16], based on the comparison of the gene expression profiles of breast cancer cells cultured in vitro in adherent and non-adherent conditions. Stem cell culture as floating monoclonal spheres was first described for normal neural stem cells [34,35], and was subsequently adopted for normal stem cells from other tissues, including breast [36]. Growth medium for stem cell expansion as spheres is commonly deprived of serum and enriched with growth factors such as epidermal growth factor (EGF) and basic fibroblast growth factor (bFGF), as well as insulin and other stimuli that favor stem cell proliferation [37]. Moreover, the growth in low-attachment flasks leads to death by anoikis of differentiated cells, while stem cells proliferate as spherical clones [37]. The expansion of the small population of CSC and its propagation in vitro is widely used to analyze the self-renewal capability of CSC and to enrich these cells from primary tumor samples and established cancer cell lines [38].

In the present study, we compared the gene expression profiles of TNBC cells grown as monolayer and their derived CSC-enriched tumorspheres. We included the murine 4T1 and the human HCC1806 cell lines in the transcriptomic analysis, then we focused our study on the genes that were differentially expressed in both cell lines. The murine breast cancer cell line was included since it represents a valuable tool for the in vivo targeting of the identified TNBC stem cells antigens. Injection of tumor cells in syngeneic mice will allow a proper interaction between the cancer and the host microenvironment cells, including those belonging to the immune system, which is fundamental to develop immune-based targeted approaches. However, to narrow our selection to genes of potential translational relevance in humans, we included in our analysis also a human TNBC cell line.

Despite their ability to form floating tumorspheres in low attachment conditions, 4T1 and HCC1806 cell lines greatly differed one from the other for what concerns CSC marker expression. Despite the CD44+/CD24− immunophenotype being suggested to identify breast CSC [39], we found that the totality of 4T1 tumorspheres and epithelial cells were strongly positive for both CD44 and CD24 expression, while almost the totality of HCC1806 were CD44 and CD24 negative. These data are in line with reports from several authors expressing concerns about the significance of these molecules as CSC markers [40,41,42]. Additionally, for CD49f (Integrin alpha 6), we found a clear difference in the expression between murine and human TNBC stem cells: while 4T1 cells were found strongly positive, with tumorspheres displaying a significantly higher expression compared to epithelial cells, both tumorspheres and epithelial cells of HCC1806 line resulted only slightly positive. Furthermore, tumorspheres from 4T1 cell line had a significantly stronger expression of the murine breast CSC marker Sca-1 compared to their epithelial counterpart. This is consistent with what we previously observed in tumorspheres from murine primary tumors and from Her2+ cell line-derived tumorspheres [19,43], which displayed an increased tumor-initiating potential compared to their epithelial, Sca-1-negative, counterpart. Finally, HCC1806 tumorspheres display a higher positivity for ALDH compared to epithelial cells. This variability in CSC marker expression between the two cell lines poses some doubts on the univocal meaning of commonly used CSC markers, evidencing a cell line- and possibly species-dependent heterogeneity that has already been reported by others [42].

As tumorspheres currently represent a widely used model in CSC studies, a deep understanding of the processes involved in their biology is of relevance for those involved in the field. Comparison of the differentially expressed transcripts in tumorspheres of 4T1 and HCC1806 cell lines allowed us to identify biological processes of interest for both murine and human TNBC stem cells. The enrichment of genes involved in cell cycle progression in the down-regulated gene set suggests that tumorspheres could be enriched in quiescent or slow cycling cells. This could be the result of a bona fide enrichment of intrinsically slow-cycling CSC and is consistent with what reported by others in tumorspheres from primary breast cancer [44] or breast cancer cell lines [45,46]. The idea that CSC may indeed be quiescent is not new, and a certain number of studies demonstrated that different cancer types contain a subset of quiescent or slow cycling CSC [47,48], thus recapitulating the features seen in normal adult tissues, where quiescent stem cells give rise to fast proliferating transiently amplifying cells that lack self-renewing potential but contribute to tissue regeneration [49]. Similar to both healthy and cancerous tissues, also breast cancer cell lines seem to retain a subpopulation of cells with stem-like properties that cycle at a slower rate compared to the bulk of the cell line [50]. Importantly, quiescence has been described as one of the several mechanisms actuated by CSC to survive chemotherapy, which preferentially targets actively proliferating cells.

In general, it is believed that an increased cholesterol and lipid biosynthesis [51,52], with cells facing a lipid metabolic reprogramming upon transformation, exerts a pro-tumorigenic role [53,54,55]. In contrast, we observed a down-regulation of transcripts coding for proteins involved in the mevalonate pathway, cholesterol metabolism and lipid biosynthesis. On the light of the fundamental lack of literature supporting a pro-tumorigenic role for decreased lipid biosynthesis, we speculate that the down-regulation of key genes involved in the mevalonate pathway and lipid/cholesterol biosynthesis in our tumorspheres may be the cause or the consequence of the cell cycle slowing, a situation in which less cholesterol and lipids are needed to support duplication. Furthermore, cholesterol has been shown to have a direct causal role in the cell cycle progression, with inhibition of cholesterol biosynthesis resulting in the blocking of cells in G_1_ [56]. Since this is pure speculation, further in vitro experiment will be needed to confirm or reject this hypothesis in our model.

Transcripts found to be up-regulated in the tumorspheres of both cell lines show to have pleiotropic functions, being the same genes involved in different biological processes. Among these, we highlight the negative regulation of apoptosis, cytokine production, response to external stimuli, regulation of signal transduction and regulation of developmental processes. This finding is in accordance with the current understanding of the CSC model, in which increased resistance to apoptosis and activation of developmental pathways are considered as hallmarks [57,58]. Overall, this study highlights the potential relevance of apoptosis resistance, CSC dormancy and cholesterol biosynthesis as pathways to be addressed to find novel approaches to fight CSC in TNBC.

Among the genes up-regulated in tumorspheres and involved in development there is TENM4. TENM4 is a large (~300 kDa) type II transmembrane glycoprotein that belongs to a family of four (TENM1 through TENM4) pair-rule proteins, showing high conservation among species and significant homology between the different family members [59]. Animal studies have demonstrated that teneurins are expressed in a highly regulated manner during embryogenesis and with differential spatial and temporal patterns in the adult central nervous system [60,61]. Besides their predominant neural distribution, teneurins mRNA have been detected in few adult organs such as the testis and thymus although at significantly lower levels [62]. Current data support the aberrant expression of teneurins in tumors of different histotypes, their involvement in cancer-related regulatory networks, their possible contribution in drug resistance [25], and their association with cancer patients’ survival [23]. However, data available from the literature reveals that the association between teneurins expression and patients’ prognosis is not univocal since it depends on the type of teneurin and on the tumor histotype [23]. As far as TENM4 deregulation in tumors is concerned, low mRNA levels in endometrial, liver, stomach and renal tumors has been associated with patients’ better survival outcome [23]. However, decreased TENM4 expression was observed in high grade serous ovarian tumors that undergo dedifferentiation [25], suggesting an opposite role for TENM4 in ovarian cancer. By investigating publicly available breast cancer patient databases, we found that TENM4 mRNA expression increases in both ductal and lobular invasive carcinomas compared to healthy breast tissue, and higher expression of TENM4 mRNA in TNBC patients is associated with a poorer RFS. This suggests that TENM4 represents a potentially useful target for TNBC. This also prompted us to further investigate how TENM4 affects stem-like properties in a murine an in a human TNBC model.

We validated TENM4 up-regulation in tumorspheres from both 4T1 and HCC1806 by RT-PCR. For this analysis, we included also other human breast cancer cell lines, such as MDA-MB-231 (TNBC), MCF7 (ER+/Her2−) and SK-BR-3 (ER−/Her2+) cells, demonstrating the up-regulation of TENM4 also in the tumorspheres derived from these cell lines. This suggests that TENM4 up-regulation could be a common feature and a possible common target of breast CSC. To evaluate whether TENM4 is only a byproduct of low-attachment growth condition or it has a causal role in the generation of tumorspheres, we silenced TENM4 through specific siRNA, observing a decrease in the number of tumorspheres in both the murine and human TNBC cell lines tested. This suggests that TENM4 is involved in CSC self-renewal, as the tumorsphere-forming assay is an in vitro surrogate of tumor-initiating potential [11]. We then sought to explore a possible mechanistic link between TENM4 expression and the observed phenotype. 

Nowadays, little is known about the mechanisms involved in TENM4 function at cellular and molecular levels. However, evidence suggest that TENM4 may act in cytoskeleton reorganization. Disruption of the *Tenm4* gene in the furue mutant mice causes inhibition of cellular process formation in oligodendrocytes, leading to small-diameter axon demyelination and subsequently causing tremors in this mutant mouse model [21]. In that study, TENM4 expression positively regulates FAK signaling, which is required for proper oligodendrocyte process formation and myelination of small diameter axons. FAK signaling pathway is also recognized to have an important role in controlling cell movement, invasion, survival, gene expression, and stem cell self-renewal in cancer cells [29]. It has been shown that TENM4 promotes filopodia-like protrusion formation in vitro [28] through FAK signaling pathway. Many data, including clinical evidence, suggest that filopodia drive cancer cell invasion [63]. Moreover, disruption of FAK in the mammary epithelium suppresses tumorigenesis in a MMTV-PyMT mouse model by depleting the pool of CSC in primary tumors, with subsequent impairment of self-renewal, migration and tumor initiating ability [64]. According to these findings, we observed an increase in total FAK protein in TNBC tumorspheres compared to epithelial cells, supporting augmented stem-like features in cells composing tumorspheres.

Targeting of TENM4 by siRNA did not lead to a decrease in total FAK protein in our models, but affected FAK phosphorylation at Y925 site, leaving Y397 phosphorylation unaffected. While most studies on the role of FAK in CSC biology focus on Y397 phosphorylation [65], the phosphorylation of Y925 is usually not investigated [66,67,68]. Nonetheless, phosphorylation at Y925 has been proposed to link FAK to the Ras pathway and to epithelial-to-mesenchymal transition (EMT), behaving as binding site for Src [69] and GRB2 [70]. A role for Y925 phosphorylation in promoting a pro-angiogenic switch in 4T1-derived tumors was also reported [71]. These findings, together with our observation that FAK phosphorylation at Y925 site is partially dependent on TENM4 expression, let us speculate that TENM4 could be linked to CSC features and metastatic progression of tumors.

Importantly, pharmacological inhibition of FAK significantly impairs 4T1 and MDA-MB-231 orthotopic tumor growth and spontaneous lung metastasis in wild type and SCID BALB/c mice, respectively [66]. All these elements led us to speculate that TENM4 could regulate migration and invasion ability of cancer cells, as well as CSC properties, through activation of FAK. Indeed, TENM4 silencing significantly impacted on the migratory ability in vitro of both murine and human TNBC cells. Moreover, it has been reported that FAK inhibition or silencing impairs the tumorsphere-forming ability of different breast cancer models, including MDA-MB-231 and other TNBC cell lines [67,72,73], and breast cancer cells from patients [74], which could explain the decreased number of tumorspheres following TENM4 silencing. Further experiments evaluating EMT and the ability to secrete pro-angiogenic factors by TNBC cells following TENM4 silencing are warranted to confirm this hypothesis in our model.

Data from a proteomic profiling revealed the existence of several TENM4-derived peptides in the urine of healthy individuals [31]. This finding suggests the existence of a secreted TENM4 form which could be used as soluble blood or urine tumor biomarker for disease detection and management. In this work, we demonstrated the presence of significantly higher amounts of TENM4 protein in the plasma from TNBC tumor-bearing mice as compared to healthy mice. Moreover, the analysis of the plasma from a little cohort of breast cancer patients and healthy donors revealed that TENM4 protein can be detected and is more abundant in patients as compared to healthy donors. Although further analyses using a larger cohort of patients are needed, our data point to the possibility of using TENM4 as a new breast cancer biomarker. Finally, the recent TENM4 protein identification on exosomes released from neuroblastoma cancer cells [32] and our data demonstrating the presence of TENM4-containing extracellular vesicles in the plasma from TNBC tumor-bearing mice and cancer patients corroborates its usefulness as cancer biomarker with potential oncogenic functions in disease progression.

## 4. Materials and Methods 

### 4.1. Human Samples

Plasma samples were obtained from the Clinical Biochemistry Laboratory, A.O.U. Città della Salute e della Scienza di Torino Hospital, Torino, Italy. Briefly, plasma specimens were prospectively collected during standard preoperative evaluation from women undergoing surgery for primary breast cancer. Leftovers from the standard diagnostic procedure were aliquoted and stored at −80 °C for further analysis. Breast cancer diagnosis was confirmed by histological examination of the corresponding surgical specimens. Subtypes were defined by immunohistochemical staining (IHC) according to St. Gallen 2013 recommendations [75], that include five categories: Luminal A, Luminal B/Her2−, Luminal B/Her2+, Her2+ and triple negative (IHC-based surrogates). IHC data were retrieved from pathology reports.

### 4.2. Mice and In Vivo Procedure

Female BALB/c and NOD/SCID-gamma null (NSG) mice were purchased from Charles River (Calco, Italy) and maintained at the Molecular Biotechnology Center, University of Turin, and treated in accordance with the University Ethical Committee and European guidelines under Directive 2010/63. In vivo experimentation was authorized by the Faculty Ethical Committee and by the Italian Ministry of Health (authorization code: 9/2018-PR). Female BALB/c and NSG mice were injected subcutaneously with 1 × 10^3^ 4T1 and 1 × 10^6^ MDA-MB-231 epithelial cells, respectively. Ranging between 30 and 40 days following 4T1 or MDA-MB-231 cell injection, blood samples were collected from anesthetized tumor-bearing and control mice via intra-cardiac puncture and supplemented with heparin solution (Sigma-Aldrich, Milano, Italy) to a final concentration of 250 U/mL to prevent coagulation. Blood samples were stored on ice and processed within 1 hour since collection. Samples were centrifuged at 4 °C, 1000× *g*, for 10 min to obtain plasma. Clear supernatant was then collected and centrifuged a second time at 4 °C, 2000× *g*, 15 min to exclude pelleted platelets. Plasma samples were stored at −80 °C until use. Primary tumors were then resected and processed for protein extraction (see Section 4.8).

### 4.3. Cell Cultures and Tumorsphere Formation

4T1 (cod. CRL-2539), HCC1806 (cod. CRL-2335), MDA-MB-231 (cod. HTB-26**)**, MCF-7 (cod. HTB-22) and SK-BR-3 (cod. HTB-30) cell lines were purchased from American Type Culture Collection (ATCC, Manassas, VA, USA). 4T1, HCC1806 and MCF-7 cells were grown in RPMI-1640 growth medium (Sigma-Aldrich, St. Louis, MO, USA) while MDA-MB-231 and SK-BR-3 were grown in Dulbecco’s Modified Eagle Medium (DMEM, Thermo-Fisher Scientific, Waltham, MA, USA). Both growth media were supplemented with 10% fetal bovine serum (FBS, Sigma-Aldrich) and Penicillin/Streptomycin solution at 1× dilution (Sigma-Aldrich). When sub-confluency was reached (approximatively every second day) cells were dissociated using trypsin-EDTA 0.5% at 1× dilution in PBS (Sigma-Aldrich) and seeded into new flasks. All the cells were periodically tested for mycoplasma contamination with the MycoAlert^TM^ Mycoplasma Detection Kit (Lonza, Basel, Switzerland). All cells used resulted to be free of mycoplasma. To generate first-passage tumorspheres, trypsin-dissociated cells were resuspended in tumorsphere growth medium at a concentration of 1.5 × 10^5^ cells/mL in Corning^®^ ultra-low attachment flasks (Sigma-Aldrich). Tumorsphere growth medium was composed of DMEM/Nutrient Mixture F-12 Ham (Sigma-Aldrich) supplemented with 0.4% bovine serum albumin (BSA, Sigma-Aldrich), 20 ng/mL bFGF (PeproTech EC Ltd., London, UK), 20 ng/mL EGF (Sigma-Aldrich) and 5 µg/mL insulin (Sigma-Aldrich). Tumorsphere growth medium was added fresh every second day. For 4T1 and HCC1806 cells, after 5 days in culture, first passage tumorspheres were enzymatically dissociated and a single-cell suspension was plated in non-adherent condition and in tumorsphere growth medium to generate second-passage tumorspheres.

### 4.4. Immuno-Staining and Flow Cytometry Analysis

Following enzymatic dissociation, cells were stained within flow cytometry tubes with AlexaFluor^®^647 anti-mouse Sca-1, PE anti-human/mouse CD44, AlexaFluor^®^488 anti-human CD24 or PE/Cy7 anti-mouse CD24, AlexaFluor^®^647 anti-human/mouse CD49f antibodies (all from BioLegend, San Diego, CA, USA) or rabbit anti-OCT4 (Abcam, Cambridge; UK), at a 1:100 dilution. Following 30 min incubation at 4 °C, cells were washed in PBS supplemented with 0.2% BSA and 0.01% sodium azide (Sigma Aldrich). Cells incubated with anti-OCT4 antibody underwent a secondary antibody staining with swine anti-rabbit FITC antibody (Dako, Santa Clara, CA, USA) at 1:50 dilution prior to acquisition. For the evaluation of ALDH activity, Aldefluor^TM^ kit (STEMCELL Technologies, Vancouver, BC, Canada) was used. Briefly, ALDH substrate was administered to cells treated or not with the ALDH inhibitor DEAB within flow cytometry tubes. Following 45 min incubation at 37 °C, 5% CO_2_, cells were rinsed in Aldefluor buffer. Cell viability was assessed by evaluating propidium iodide (Sigma Aldrich) incorporation. All data were acquired through CyAn ADP flow cytometer (DakoCytomation, Beckman Coulter, Brea, CA, USA) and data were analyzed using Summit 4.3.02 Build software (Beckman Coulter).

### 4.5. Total RNA Extraction and RNA Sequencing

Total RNA was isolated from 2 × 10^6^ epithelial and tumorsphere-derived cells from 4T1 and HCC1806 cell lines using the TriZol reagent (Thermo-Fisher Scientific) and following manufacturer’s instructions. Biological triplicates for each condition were used. Genomic DNA contaminations were removed from RNA samples with the Ambion^®^ DNA-free kit (Thermo-Fisher Scientific). RNA concentration and quality were estimated with NanoVuePlus Spectrophotometer (GE Healthcare, Little Chalfont, UK) and Agilent 2100 Bioanalyzer (Agilent Technologies, Santa Clara, CA, USA), respectively. Libraries for RNA sequencing were generated using TruSeq RNA sample preparation kit v2 (Illumina Inc., San Diego, CA, USA) following manufacturer’s instructions, using 1 µg of total RNA as input material and 15 PCR cycles for DNA amplification. Libraries were quantified by Qubit 2.0 Fluorometer using ds DNA High Sensitivity Qubit Assay (Thermo-Fisher Scientific) and pooled together in equimolar amounts. Cluster generation was performed using a cBot System (Illumina Inc.). Libraries were sequenced with an Illumina HiSeq 1000 sequencer (Illumina Inc.) generating 100-bp paired-end sequences.

### 4.6. RNAseq Data Analysis

The quality of RNAseq data was evaluated with FastQC tool (Babraham Institute, Cambridge, UK; http://www.bioinformatics.babraham.ac.uk/projects/fastqc/ version 0.11.6). Coding RNAs were quantified mapping reads against UCSC database. Differential expression analysis was performed with limma [76] using voom normalization and BH procedure for p value correction [77]. The genes with a Log_2_FC ≥ 1 or Log_2_FC ≤ −1 and adjusted *p*-value ≤ 0.1 were considered as differentially expressed between tumorpsheres and the corresponding epithelial counterpart. The resulting datasets were then loaded on Ingenuity Pathway Analysis (IPA, Qiagen, Chatsworth, CA, USA) and differentially expressed transcripts in common between mouse 4T1 and human HCC1806 cell lines were considered for further analysis. This allowed the generation of down-regulated and up-regulated genesets. The datasets are available in the Gene Expression Omnibus (GEO) repository, Accession code GSE109798 (https://www.ncbi.nlm.nih.gov/geo/query/acc.cgi?acc=GSE109798, accessed on 30 January 2018).

### 4.7. Gene Ontology Analysis

Down-regulated and up-regulated genesets were separately entered into the Gene Ontology (GO) enrichment analysis tool available at the Gene Ontology Consortium website (http://www.geneontology.org/page/go-enrichment-analysis, accessed on 23 July 2017) and analyzed through the PANTHER Overrepresentation Test (release version 2016/07/15) using the *Homo sapiens* reference list (all genes in the GO database released on 2017/02/28). Gene ontology option “GO biological process complete” was selected and a functional annotation chart was generated. A maximum *p*-value of 0.05 was chosen to select only significant categories.

### 4.8. Meta-Analysis on Patient Databases

Univariate survival analysis was performed using the Kaplan-Meier Plotter free software [26] (Semmelweis University, Budapest, Hungary, http://kmplot.com/analysis/index.php?p=service&cancer=breast, accessed on 15 March 2017). RFS data were presented as Kaplan-Meier plots and tested for significance using log-rank tests. Patients were stratified by expression of the gene of interest. To define the cutoff between high and low expression “auto select best cutoff” function embedded in the Kaplan-Meier Plotter website was used. Briefly, this algorithm determines the optimal cutoff for maximum significance between “high expression” versus “low expression” arms by calculating all percentiles between the lower and upper quartiles and automatically selecting the best expression threshold as the cutoff value. Regarding TENM4 (Affymetrix ID 213273), the cutoff value used was 177, and the expression range of the probe was 5–820. In case the gene of interest had more than one microarray reporter available (Affymetrix ID), the mean expression of all available probes for a given gene was used. Analysis was restricted to samples displaying negative ER, PR and Her2 status, selected by ticking the appropriate option in the Kaplan-Meier Plotter software.

The relative mRNA expression of TENM4 in human breast tumor samples was determined by querying the Oncomine database (version 4.5; https://www.oncomine.org/ accessed on 15 March 2017). TENM4 mRNA expression was queried in The Cancer Genome Atlas (TCGA) breast cancer dataset (released 2011/09/02) investigating the Agilent IDs A_23_P47746, A_24_P342309 and A_24_P342312. Oncomine output data were sorted to isolate associations between lobular or ductal invasive carcinoma and normal breast tissue and reported as the log2 median-centered ratio using box-and-whiskers plots (dots: maximum and minimum values; whiskers, 90/10 percentiles; box, 75/25 percentiles; line, median of all samples).

### 4.9. RT-PCR

DNA-free RNA was prepared as reported above in the RNA extraction section. cDNA was obtained by 1 µg of RNA retro-transcribed with Ambion^®^ RETROscript reagents (Thermo-Fisher Scientific). Target mRNA was amplified through real-time PCR using gene-specific primers (QuantiTect Primer Assay; Qiagen) and SYBR green PCR Master Mix (Applied Biosystems). A 7300 RT-PCR system (Applied Biosystems) was used to perform the real-time PCR and the Applied Biosystems SDS Software Version 1.3.1 was used to analyze data. Quantitative normalization was performed on the expression of GAPDH. The tumorspheres transcripts expression levels relative to epithelial cells were calculated using the comparative ΔCt method [78].

### 4.10. RNA Interference

Tumorspheres or epithelial cells were transfected with either the TENM4 targeting or control siRNA (100 nM) using Lipofectamine 2000 (Invitrogen) for twenty-four hours. Mouse TENM4-specific siRNA: ON-TARGETplus siRNA Pool (FE5L062216010005 Dharmacon Inc, Lafayette, CO, USA); mouse siRNA negative control: ON-TARGETplus Non-targeting Control Pool (FE5D0018101005 Dharmacon); human TENM4-specific siRNA: Ambion Silencer Select (4392420 Ambion, Thermo-Fisher Scientific); human siRNA negative control: Ambion Silencer Negative Control #1 (4390843 Ambion Thermo-Fisher Scientific)**.** Epithelial or tumorsphere-derived single cell suspension were transfected following Lipofectamine manufacturer’s instruction in a 6-well plate (a Corning^®^ Ultra-Low attachment 6-well plate, Thermo-Fisher Scientific, was used for tumorspheres transfection with appropriate antibiotic-free medium). Twenty-four (MDA-MB-231 and HCC1806) or 48 (4T1) hours after transfection, TENM4 silenced epithelial cells or tumorspheres were harvested and used for migration assays, frozen for Western blot or counted to assess the tumorsphere-generation ability.

### 4.11. Immunoblotting

Frozen or fresh cells, not previously treated with trypsin, were incubated in RIPA buffer (150 mM NaCl; 50 mM Tris-HCl pH 8.00; Sodium dodecyl sulphate (SDS) 0.1%; Sodium Deoxycholate, 0.5%; Nonidet P-40, 1%) supplemented with NaVO_4_, NaF, PMSF and protease inhibitors cocktail (Sigma-Aldrich) for 40 min on ice. Cell lysates were centrifuged 5 min at 12.000 *rpm* and the protein-containing supernatant was harvested for use. Regarding tumor samples collected from mice, they were homogenized using Turrax homogenizer (IKA, Staufen, Germany) in RIPA buffer. Subsequent protein extraction was performed as for cell lines. Total protein concentration was quantified using the Pierce™ BCA Protein Assay Kit (Thermo-Fisher Scientific). Following 5′ denaturation at 95 °C in 2-Mercaptoethanol-containing Laemmli Sample Buffer (Bio-Rad, Hercules, CA, USA), equal amounts of protein (ranging between 30 and 70 µg) were separated through electrophoresis in a 4–20% Mini-Protean TGX precast gel (Bio-Rad) and then transferred onto an Immobilion-P PVDF membrane (Merck Millipore, Billerica, MA, USA). Following blocking with 5% non-fat dry milk (Santa Cruz Biotechnology, Dallas, TX, USA) or 5% BSA (Sigma-Aldrich) in wash buffer (Tris Buffered Saline - TBS-supplemented with 0.05% Tween-20 from Sigma-Aldrich), the membrane was incubated overnight at 4°C with sheep anti-TENM4 (1 µg/mL, Cat#AF6320, R&D Systems, Minneapolis, MN, USA), mouse anti-total FAK (1:1000, Cat#AHO1272, Thermo-Fisher Scientific), rabbit anti-pY925 FAK (1:1000, Cat#3283, Cell Signaling Technology), mouse anti-Vinculin (1:8000, produced in house) or mouse anti-β-Actin (1:200, Clone AC-15, Santa Cruz Biotechnology) antibodies in the proper blocking buffer. Membrane was then rinsed and incubated 1 hour at room temperature with HRP-conjugated anti-sheep (1:1000, Cat#HAF016, R&D Systems), anti-mouse (1:2000, Cat#A4416, Sigma-Aldrich) or anti-rabbit (1:2000, Cat#A0545, Sigma-Aldrich) antibody in blocking buffer. Vinculin or β-actin were used as loading control. Membranes were incubated with Pierce^®^ ECL Western Blotting Substrate (Thermo Fisher Scientific) and images were acquired using a ChemiDoc™ Touch Imaging System (Bio-Rad). Peak area was computed using Fiji-ImageJ software (National Institute of Health, Bethesda, MD, USA, https://imagej.nih.gov/ij/index.html).

### 4.12. Tumorsphere-Generation Assay

To assess the clonogenic potential of cells in low-attachment conditions, 4T1 and HCC1806 first passage tumorspheres and MDA-MB-231 epithelial single cell suspensions were seeded in antibiotic-free tumorsphere growth medium at a concentration of 6 x 10^4^ cells/mL in a Corning^®^ Ultra-Low attachment 6-well plate (Thermo Fisher Scientific) and treated as reported above in Section 4.10. Twenty-four hours after treatment, tumorspheres were harvested and 400 µL of tumorsphere suspension were distributed dropwise in a 96-well plate. The number of tumorspheres per well were counted after 2 (MDA-MB-231) or 5 (4T1 and HCC1806) days, through an optical microscope at 400x and summed, then the total number of tumorspheres was calculated and the ratio of tumorspheres generated per 1 × 10^4^ cells seeded was inferred with the following formula:

$$X=\frac{\left(Total\;number\;of\;tumorspheres\right)\times 10^{4}}{Total\;number\;of\;cells\;initially\;plated}$$

Data have been expressed as percentage of reduction in tumorspheres forming ability and are the results of at least three independent experiments. Phase-contrast microscopy images of the plates containing spheres were acquired using the Apotome Zeiss microscope (Olympus Corp., Tokyo, Japan) using the10× objective lens, and analyzed using Fiji-ImageJ Software.

### 4.13. Cell Migration Assay

After TENM4 silencing, dissociated 4T1 and HCC1806 tumorspheres and MDA-MB-231 epithelial single cell suspension were seeded at a concentration of 2 × 10^5^ cells/mL in 100 μL of serum-free medium in the top chamber of a 24-Transwell plate (8-μm pore size; Corning, Amsterdam, The Netherlands). The bottom chambers of the Transwell plates were filled with 10% FBS-supplemented medium (600 μL per well) and cells were incubated at 37 °C in a 5% CO2 atmosphere for additional 48 (4T1) or 72 (MDA-MB-231) hours. Then, the non-migrated cells on the top side of the filter were removed by scrubbing with cotton tipped swab. Migrated cells on the bottom side of the filter were fixed with 2.5% glutaraldehyde (Sigma-Aldrich) and stained with 0.2% crystal violet (Sigma-Aldrich). After washing, the migrated cells of five randomly selected fields per well were imaged using an Olympus BX41 microscope (Olympus Corp., Tokyo, Japan) and analyzed using Fiji and Imagej Softwares (Rasband, W.S., ImageJ, U. S. National Institutes of Health). Averaged data from at least three independent experiments were used.

### 4.14. TENM4 Enzyme-Linked Immunosorbent Assay (ELISA)

To evaluate the level of TENM4 protein in the plasma collected from 4T1- and MDA-MB-231-tumor bearing mice, healthy donors and breast cancer patients, the Human Tenascin M4 or Mouse Tenascin M4 ELISA kits (both from MyBioSource, San Diego, CA, USA) were used following themanufacturer’s instructions. Briefly, 50 μL of undiluted plasma were incubated 1 hour at 37 °C on a ready-to-use ELISA plate pre-coated with anti-TENM4 antibodies. The horseradish peroxidase (HRP) enzyme-based colorimetric detection system was exploited to reveal TENM4 antigen in the samples, measuring the optical density (O.D.) at 450 nm wavelength with an ELISA plate reader (Bio-Rad). The concentration gradients of the kit standards were used to generate a standard curve and a professional curve fitting software (GraphPad, San Diego, CA, USA) was applied to interpolate the O.D. values and to determine the concentration of TENM4 in each sample.

### 4.15. Extracellular Vesicles (EVs) Isolation and Characterization

Conditioned media (100 mL) were collected from 4T1 and MDA-MB-231 cells grown at sub-confluence for 48 hours without FBS and used for EVs isolation through the Exo-spin Exosome Purification kit (Cell Guidance Systems, Cambridge, UK) according to the manufacturer’s instructions. Briefly, cell debris and large vesicles in the media were removed by differential centrifugations, a first one at 300× *g* for 10 min followed by a second one at 16,000× *g* for 30 min. To precipitate the exosome-containing fractions, the supernatants were mixed with the Exo-spin™ Buffer in a 2:1 ratio, incubated 1 hour at 4 °C and then centrifuged at 16,000× *g* for 1 hour. The EVs-containing pellets were resuspended in 100 μL of PBS and EVs were purified using the Exo-spin™ columns. To precipitate the EVs from plasma collected from healthy donors, breast cancer patient and tumor-bearing mice, 250 uL of plasma was thawed on ice and 63 uL of Exoquick^TM^ (System Biosciences) was added. After an overnight incubation, the mixture (plasma/exoquick) was centrifuge at 1500× *g* for 30 min at 4 °C and the resulting pellet was resuspended in 200 uL of PBS. The EVs suspension was diluted in PBS and visualized on the Nanosight LM10 instrument (Particle Characterization Laboratories, Novato, CA, USA). The particle size profile and concentration in plasma samples were evaluated with nanoparticle tracking analysis (NTA) 3.2 software (Malvern Panalytical, Egham, UK). After characterization, purified EVs were lysed for immunoblot, as previously described for fresh and frozen tumor cells, in order to detect the presence of TENM4 (see Section 4.11). Rabbit anti-CD9 and anti-CD63 antibodies were used as EVs controls (1:1000, ExoAb Antibody kit, System Biosciences, Embarcadero Way, Palo Alto, CA, USA) following manufacturer’s instruction. A mouse anti-CD9 antibody was used as EVs control for human samples (1:1000, 5G6 clone, Thermofisher).

### 4.16. Statistical Analysis

Differences of gene expression assessed by real-time PCR, tumorsphere generation potential, migration ability and TENM4 amount in the sera of tumor-bearing mice and breast cancer patients were evaluated using an unpaired *Student’s t*-test. RFS of patients was assessed through a log-rank test. Data are shown as means ± SEM unless otherwise stated. Values of *p* < 0.05 were considered significant unless otherwise stated. 

## 5. Conclusions

TNBC is still considered as a single clinical entity, uniformly treated with chemotherapy, even if high-throughput approaches revealed a high degree of heterogeneity from the molecular point of view [3,79,80]. In the current work, a major limitation is of course the use of only two human TNBC cell lines, the mesenchymal stem-like MDA-MB-231 and the basal-like 2 HCC1806 cells [80], which cannot obviously recapitulate the diversity of this disease. Nonetheless, this work highlighted a restricted number of potential targets that can be further investigated in the large plethora of existing human breast cancer cell lines belonging to the different molecular subtypes that collectively represent the TNBC complexity [80]. Furthermore, inclusion of the murine cell line 4T1 in our study will allow the development of preclinical strategies that include active immunotherapy, this last approach not being possible in xenotransplantation models. Among the genes up-regulated in tumorspheres and coding for transmembrane proteins, we focused on TENM-4, showing that its up-regulation could be a common feature of tumorspheres of multiple cell lines and that its role in the CSC-self renewal and cell migratory ability could be mediated by FAK. The use of TNBC cell lines in which TENM4 is only transiently silenced because of the use of siRNA technology pones some limitations among which the impossibility to study the consequences of TENM4 silencing in vivo. Indeed, time course analysis of TENM4 mRNA and protein expression in both 4T1 and MDA-MB-231 epithelial and tumorsphere-derived cells clearly demonstrate that significant TENM4 silencing is achieved between 24 and 48 hours from siRNA transfection but then progressively disappeared. Further in vivo experiments, which in this study are lacking, using stably TENM4 knock out murine TNBC cells that we have recently obtained by exploiting the CRISPR/Cas9 technology will help us to better define the role of TENM4 on tumor initiation and progression.

## Figures and Tables

**Table 1:** Up-regulated transcripts encoding for transmembrane proteins.

Gene Name	Protein Full Name	Log_2_FC	
		4T1	HCC1806
ACVR2B	*Activin receptor type-2B*	1.748951	1.098227
AGRN	*Agrin*	1.871666	1.405154
B2M	*Beta-2 Microglobulin*	1.942163	1.50054
COMTD1	*Catechol-O-Methyltransferase Domain Containing 1*	1.514779	1.281886
F11R	*Junctional adhesion molecule A*	2.138065	2.006326
FAS	*Fas Cell Surface Death Receptor*	2.016033	1.945283
FZD7	*Frizzled-7*	1.672478	1.705518
IGF1R	*Insulin-like growth factor 1 receptor*	1.090656	1.087368
IGSF8	*Immunoglobulin Superfamily, Member 8*	1.429017	1.159
IL6ST	*Interleukin 6 Signal Transducer*	1.518154	1.642875
ITGA10	*Integrin Alpha 10*	2.02747	1.552955
LGALS3BP	*Lectin, Galactoside-binding, Soluble, 3 Binding Protein*	2.217845	1.546496
PIP5K1C	*Phosphatidylinositol 4-phosphate 5-kinase type-1 gamma*	1.301736	1.066668
PMP22	*Peripheral Myelin Protein 22*	2.157392	1.797665
SCARA5	*Scavenger Receptor Class A, Member 5*	4.096917	1.486333
SSC5D	*Scavenger Receptor Cysteine Rich Family, 5 Domains*	1.662582	2.080388
TENM4	*Teneurin 4*	2.862506	2.421285

## Data Availability

Not Applicable.

## Supplementary Materials

The following are available online at https://www.mdpi.com/2072-6694/13/4/894/s1, Table S1: Up-regulated genes in tumorspheres, Table S2, Down-regulated genes in tumorspheres, Figure S1: Correlation between gene expression and patients’ prognosis, Figure S2: Characterization of MDA-MB-231 cells and derived tumorspheres, Figure S3: TENM4 expression in breast cancer and in plasma from patients, Figure S4:TENM4 role in HCC1806 CSC self renewal and migratory ability, Figure S5: Effect of TENM4 silencing on CSC markers expression in murine and human TNBC stem cells; original blot pictures.
