# Supplementary material for: Identification of TENM4 as a Novel Cancer Stem Cell-Associated Molecule and Potential Target in Triple Negative Breast Cancer
Source: Cancers (Basel). 2021 Feb 20;13(4):894. doi: 10.3390/cancers13040894 (PMC7924390; doi:10.3390/cancers13040894)

Supplementary material

Identification of TENM4 as a novel Cancer Stem Cell-associated molecule and potential target in Triple Negative Breast Cancer

Roberto Ruiu, Giuseppina Barutello, Maddalena Arigoni, Federica Riccardo, Laura Conti, Giulia Peppino, Laura Annaratore, Caterina Marchiò, Giulio Mengozzi, Raffaele Adolfo Calogero, Federica Cavallo and Elena Quaglino.

In this section the raw colorimetric image of each PVDF membrane/blot with the transferred protein ladder, the ECL image and the superimposition between the colorimetric (containing the marker bands) and the ECL image to see the protein of interest and the ladder at the same time are reported. The lanes in the black rectangles are those of the samples shown in the figures of the manuscript.

For the current study the ThermoScientific (Rodano; Milan, Italy) PageRuler™ Prestained Protein Ladder ranging from 10 kDa to 170 kDa (cat#26616) and that ranging from 10 to 250 KDa (cat#26619) were used.

Densitometric analyses have been carried out by using Image Lab software (BioRad; Segrate; Milan, Italy). Densitometric values are shown at the end of each lane in the image showing the protein of interest and the ladder. For each blot we chose a reference lane that we marked as “100”. All the other bands have been normalized accordingly.

A) W. Blot Fig. 4b; left panel (4T1), and central panel (HCC1806), for **TENM4** and **Vinculin**. Samples in black rectangles are shown.

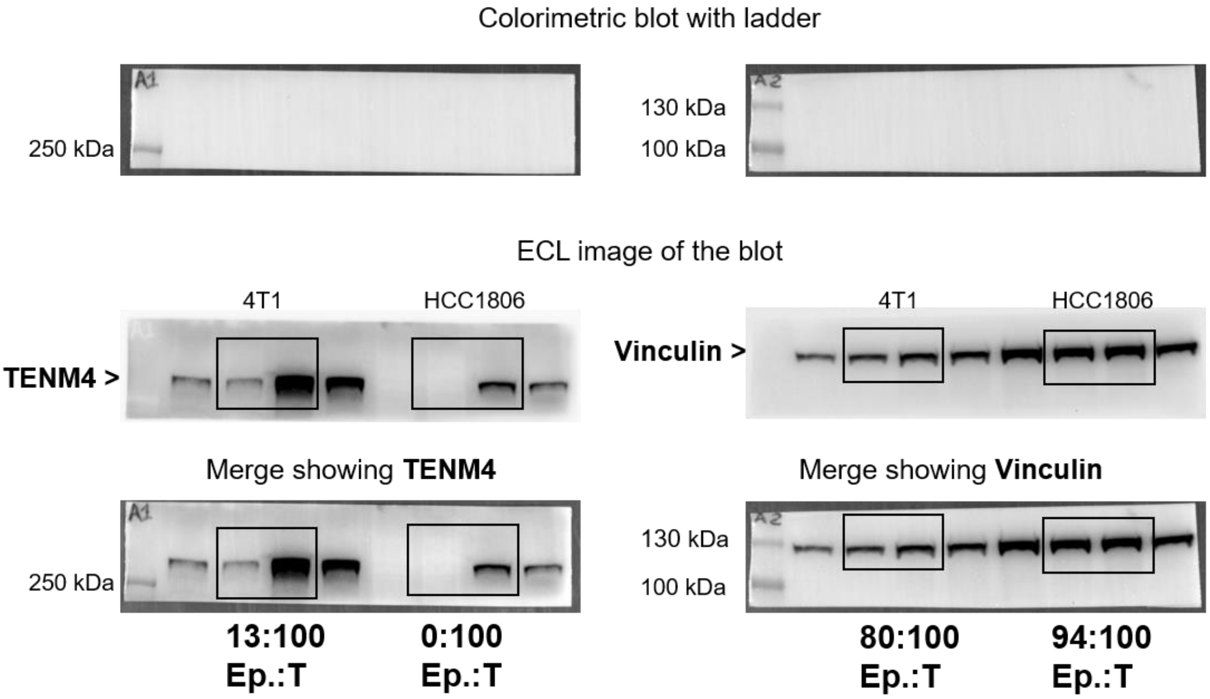

B) W. Blot Fig. 4b; left panel (4T1), and central panel (HCC1806), for **FAK**. Samples in black rectangles are shown.

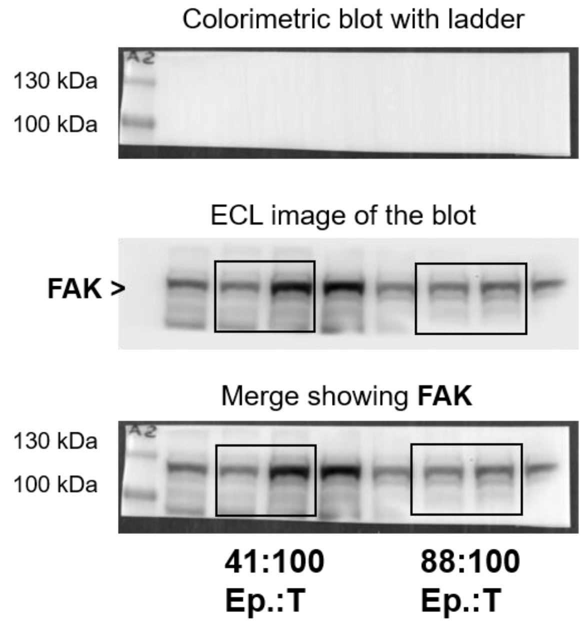

A) W. Blot Fig. 4b; right panel (MDA-MB-231); for **TENM4** and **β-actin**. Samples in black rectangles are shown in the fugure.

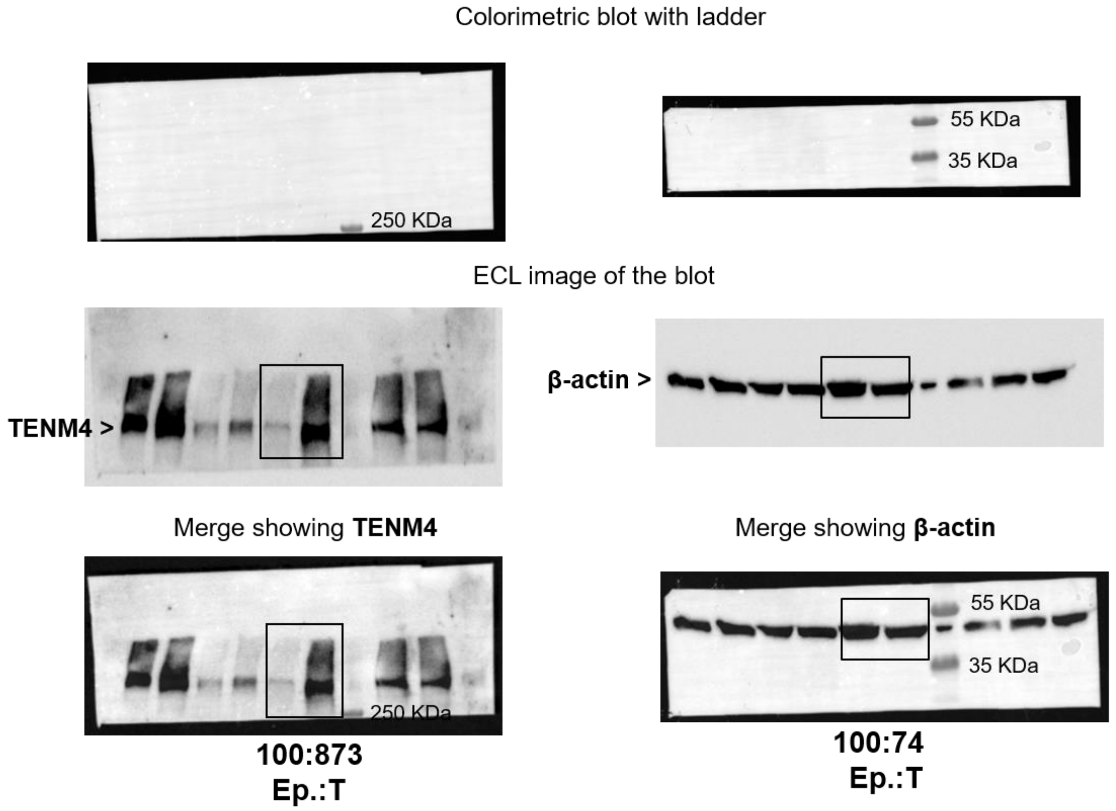

B) W. Blot Fig. 4b; right panel (MDA-MB-231); for **FAK**. Samples in black rectangles are shown in the fugure.

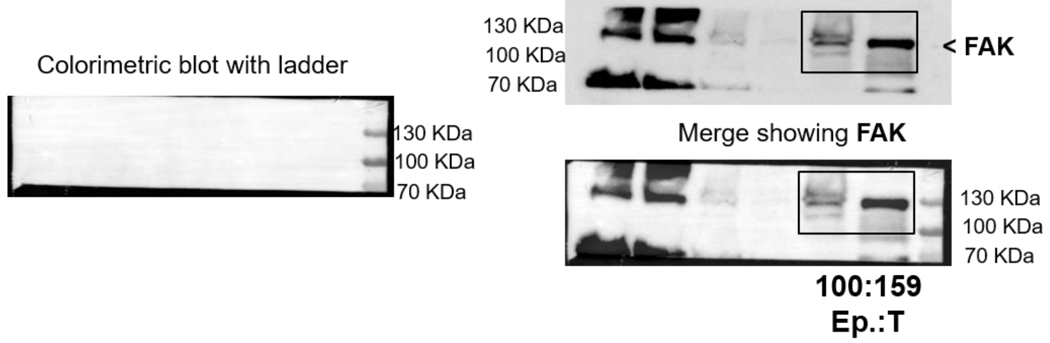

A) W. Blot Fig. 4c for **TENM4** and **Vinculin**. Samples in black rectangles are shown in the fugure.

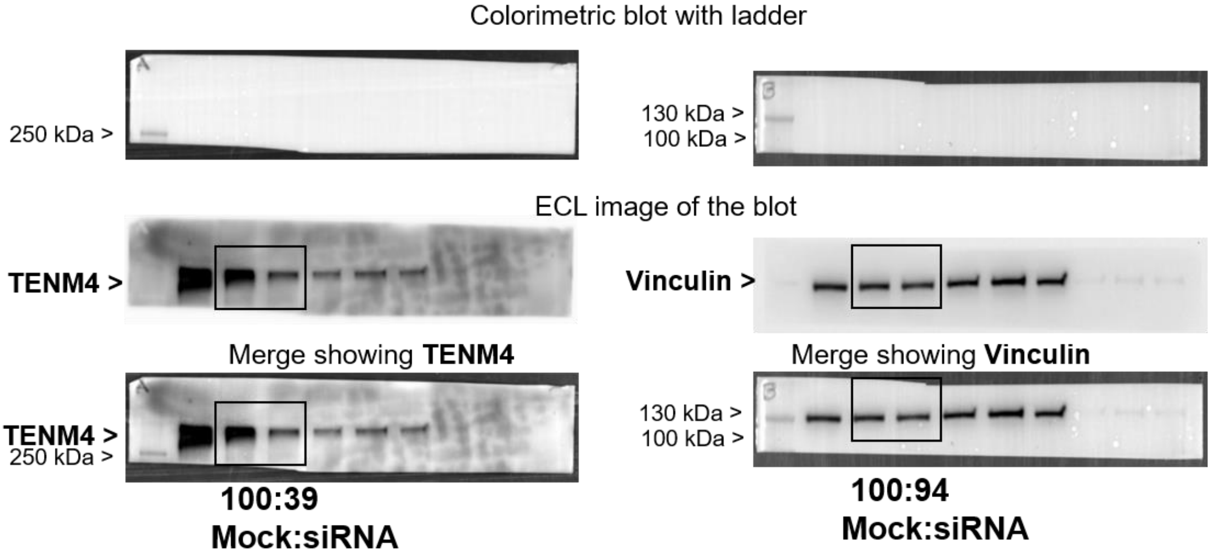

B) W. Blot Fig. 4c for **FAK** and **pFAK^{Y925}**. Samples in black rectangles are shown in the fugure.

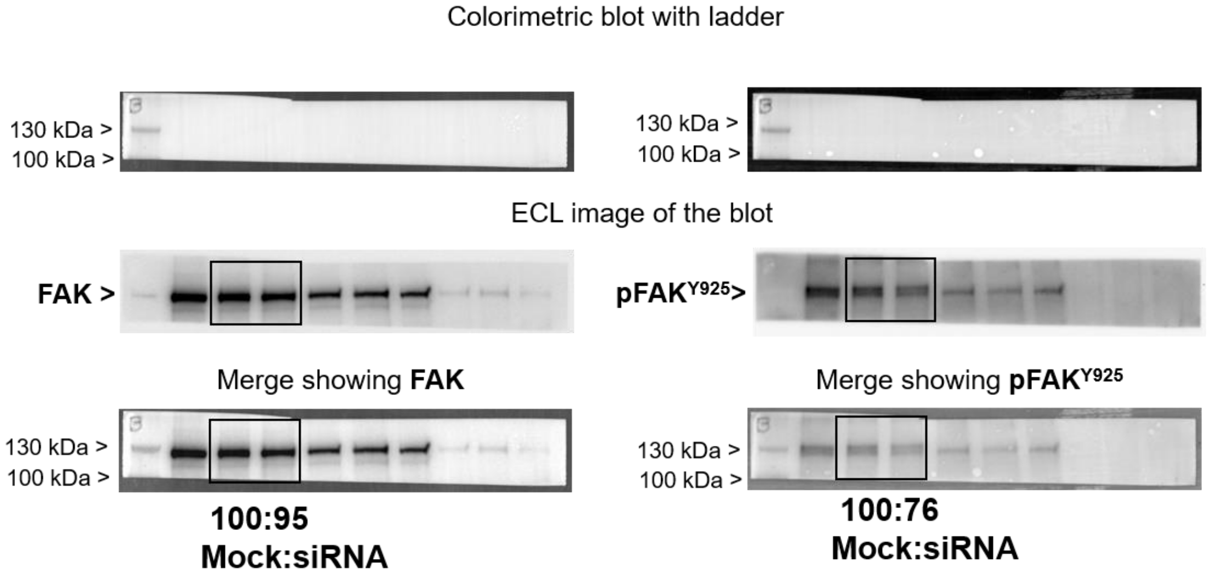

A) W. Blot Fig. 4f for **TENM4** and **β-actin**. Samples in black rectangles are shown in the fugure.

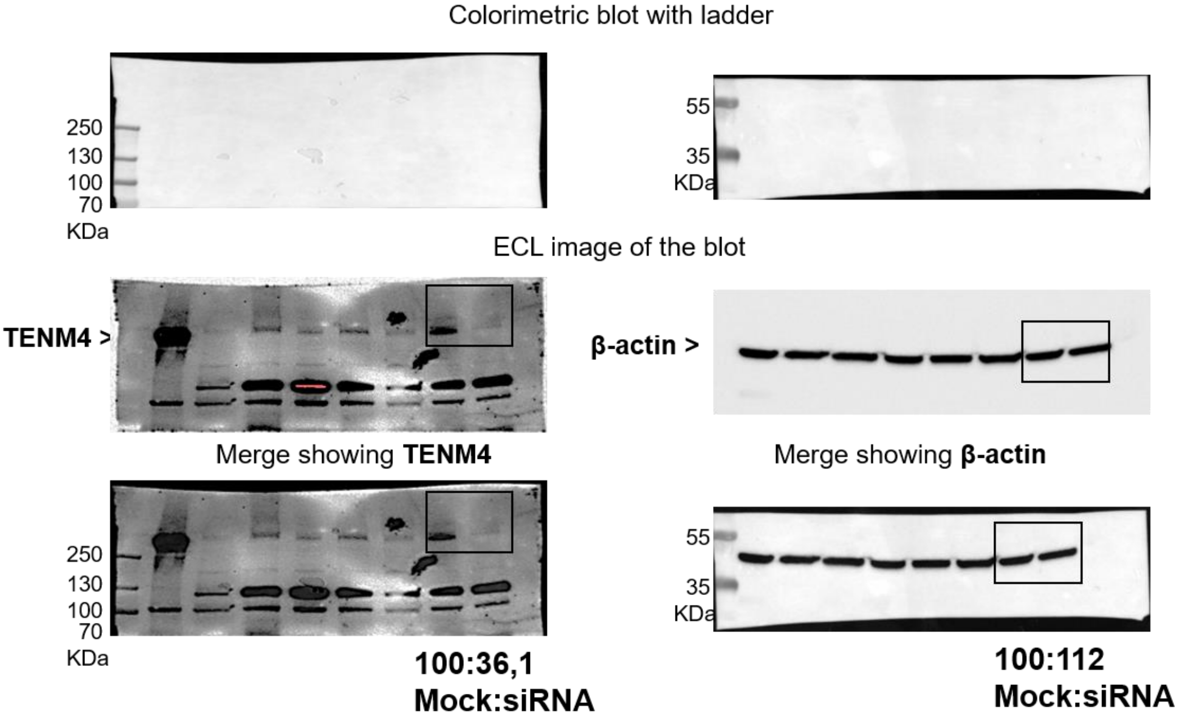

B) W. Blot Fig. 4f for **FAK** and **pFAK^{Y925}**. Samples in black rectangles are shown in the fugure.

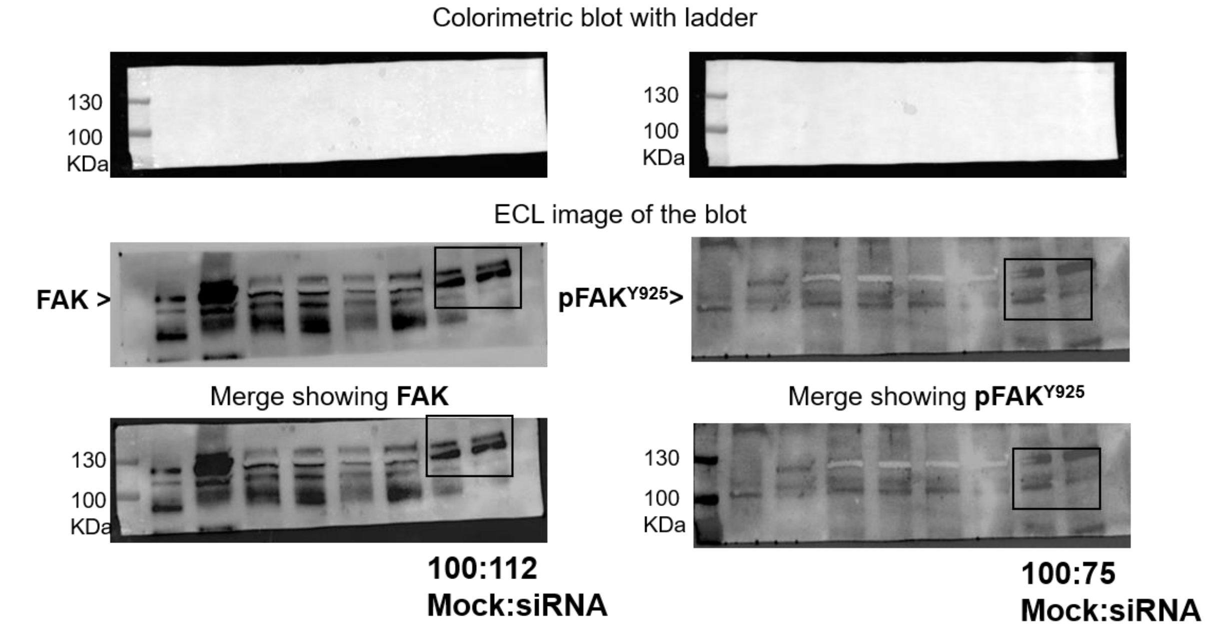

W. Blot Figure 5a (4T1; upper panel) for **TENM4** and **β-actin**. Samples in black rectangles are shown)

Colorimetric blot with ladder

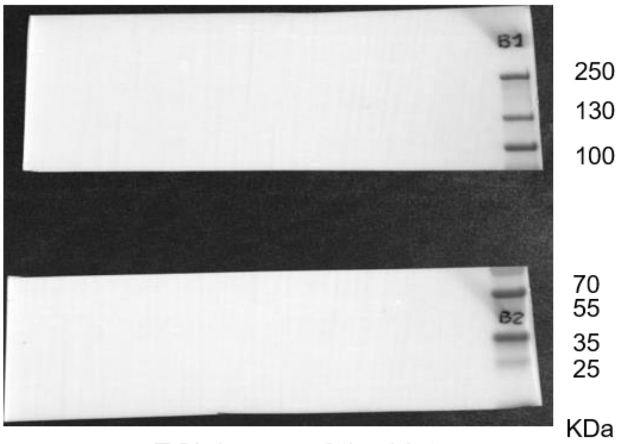

ECL image of the blot

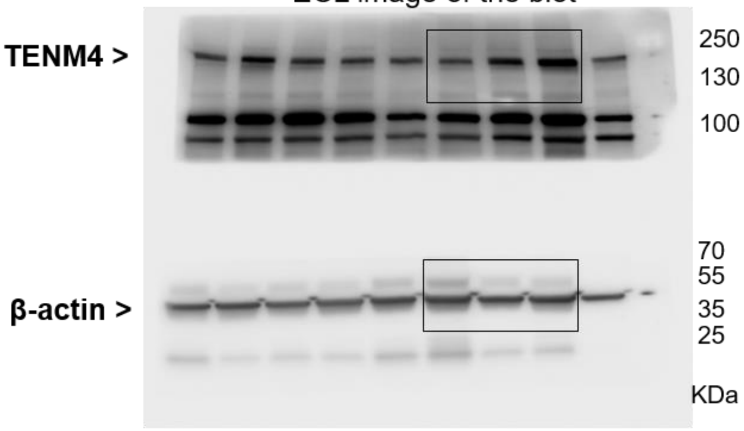

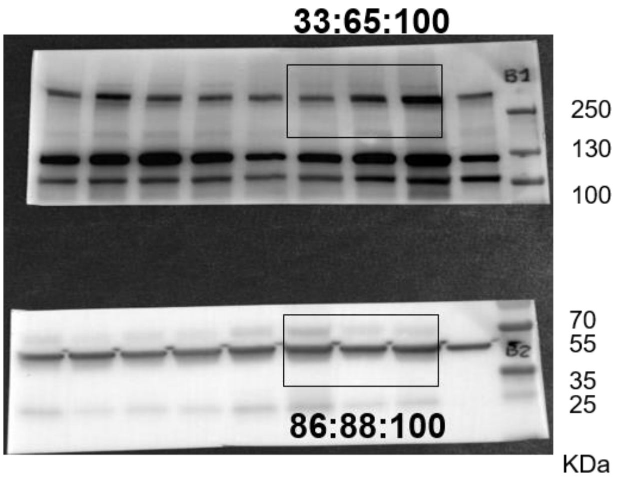

Merge showing
TENM4

or

β-actin

W. Blot Figure 5a (MDA-MB-231; lower panel) for **TENM4** and **β-actin**. Samples in black rectangles are shown).

Colorimetric blot with ladder

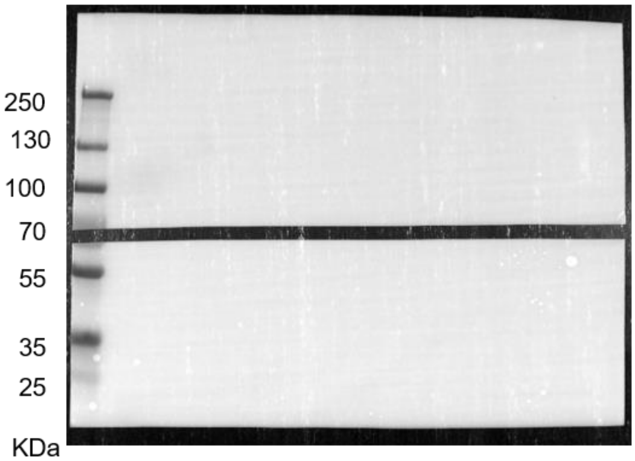

ECL image of the blot

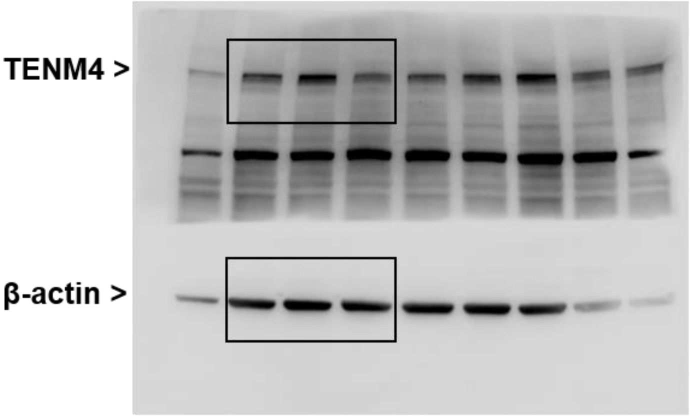

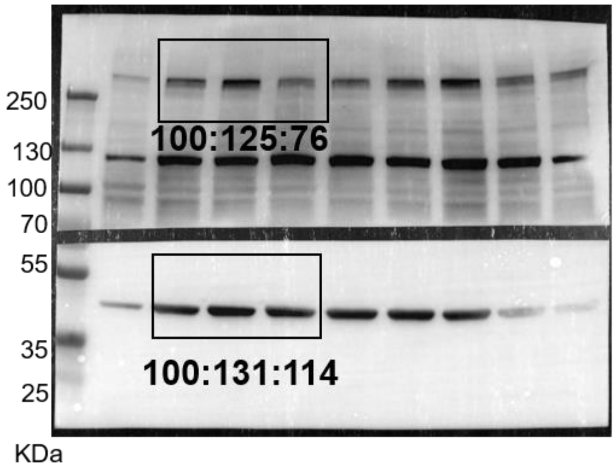

Merge showing **TENM4**

or

β-actin

A) W. Blot Figure 5f, for **TENM4**. Samples in black rectangles are shown.

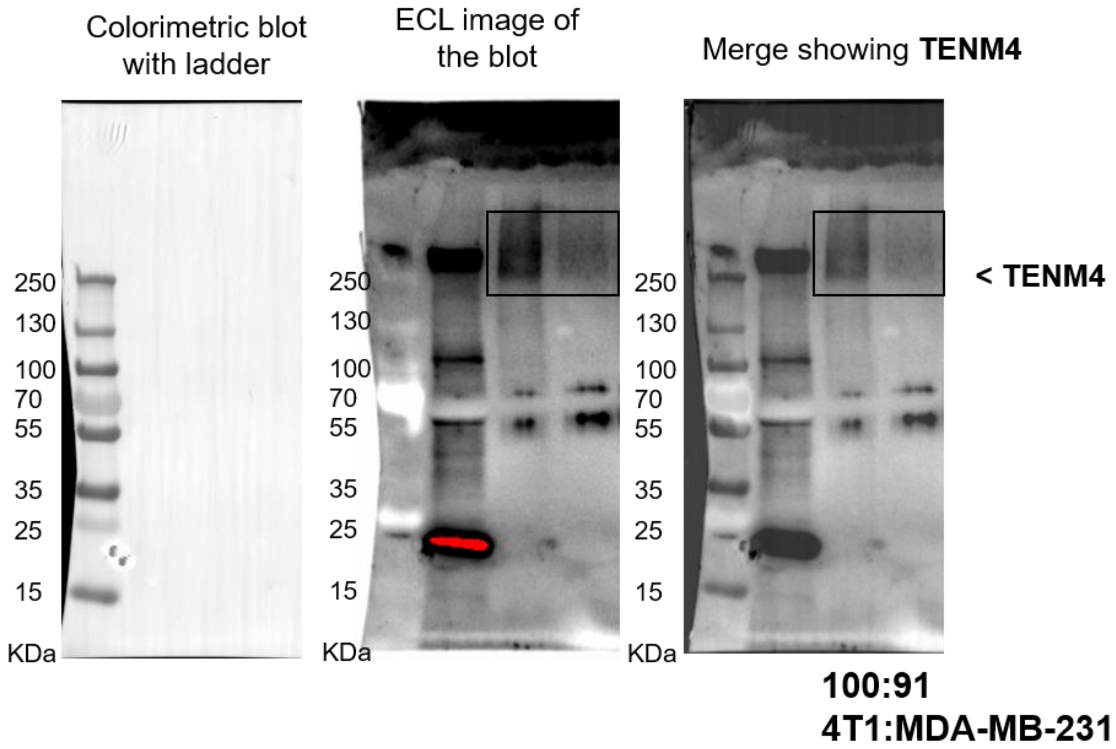

B) W. Blot Figure 5f, for **CD9**. Samples in black rectangles are shown.

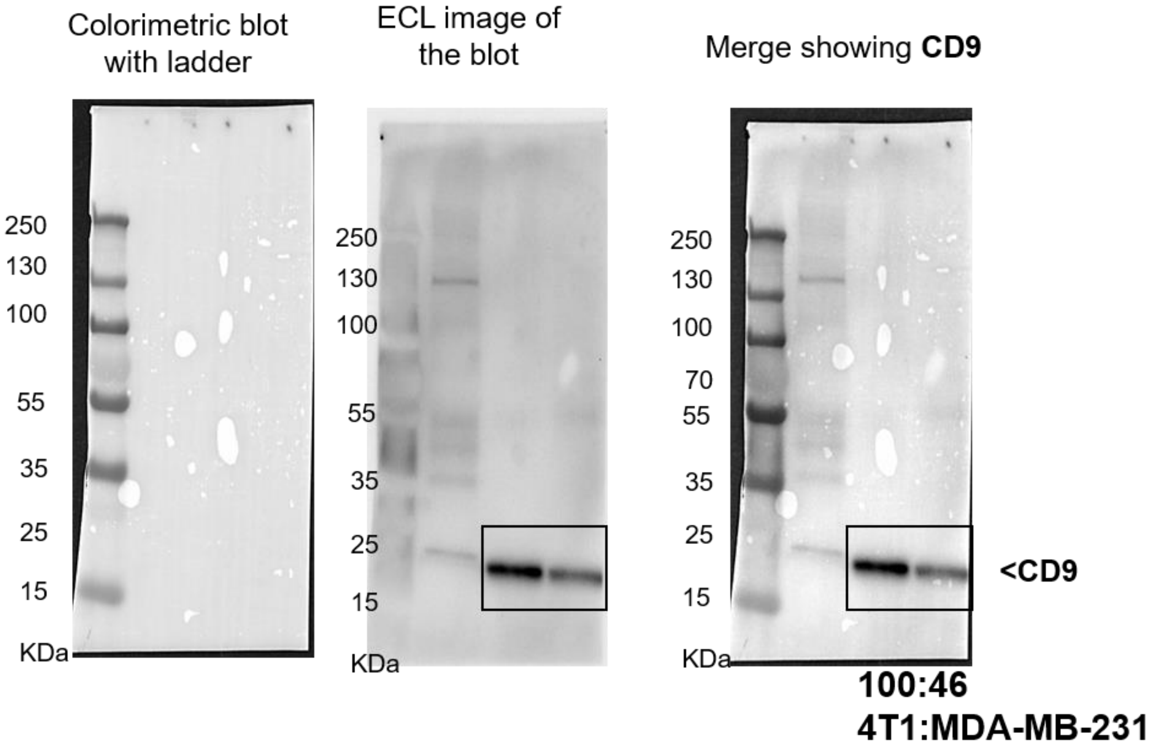

A) W. Blot Figure 5g; left panel (4T1), and central panel (MDA-MB-231) for **TENM4**. Samples in black rectangles are shown.

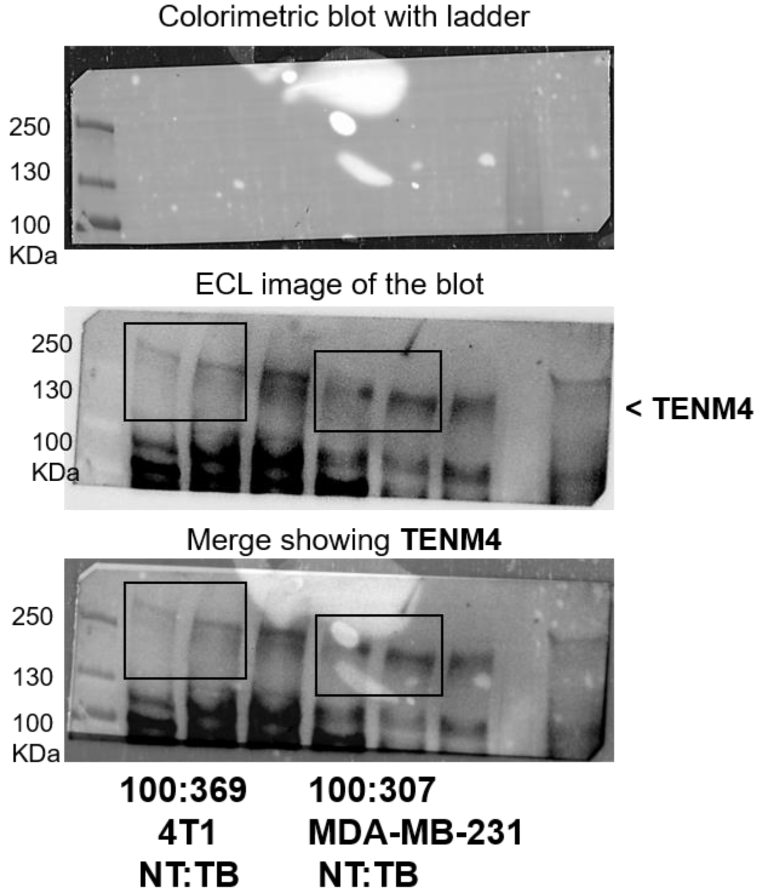

B) W. Blot Figure 5g; left panel (4T1), and central panel (MDA-MB-231) for **CD9**. Samples in black rectangles are shown.

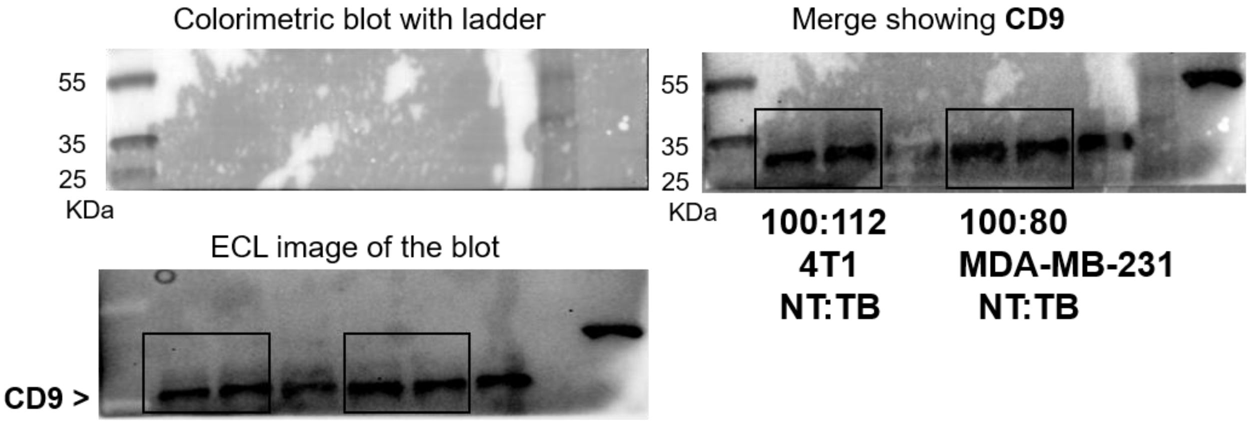

A) W. Blot Figure 5g; right panel (patients), for **TENM4**. Samples in black rectangles are shown.

Colorimetric blot with ladder

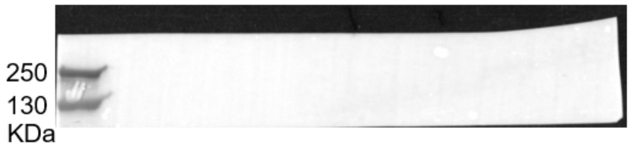

ECL image of the blot

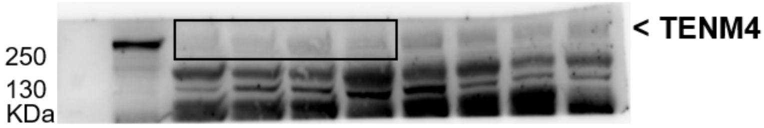

Merge showing **TENM4**

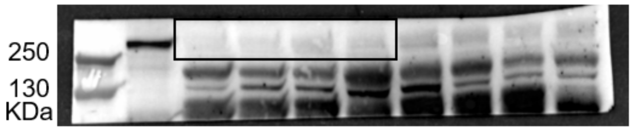

100:85:142:114
HD:HD:TNBC:TNBC

B) W. Blot Figure 5g; right panel (patients), for **CD9**. Samples in black rectangles are shown.

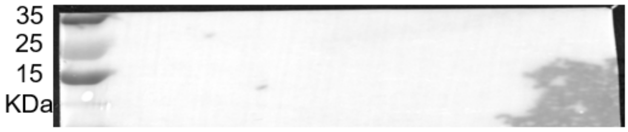

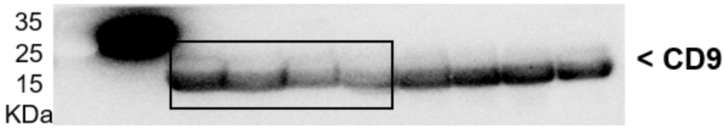

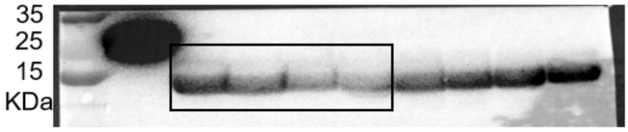

100:97:53:59
HD:HD:TNBC:TNBC

A) W. Blot Figure S3b, for **TENM4**. Samples in black rectangles are shown.

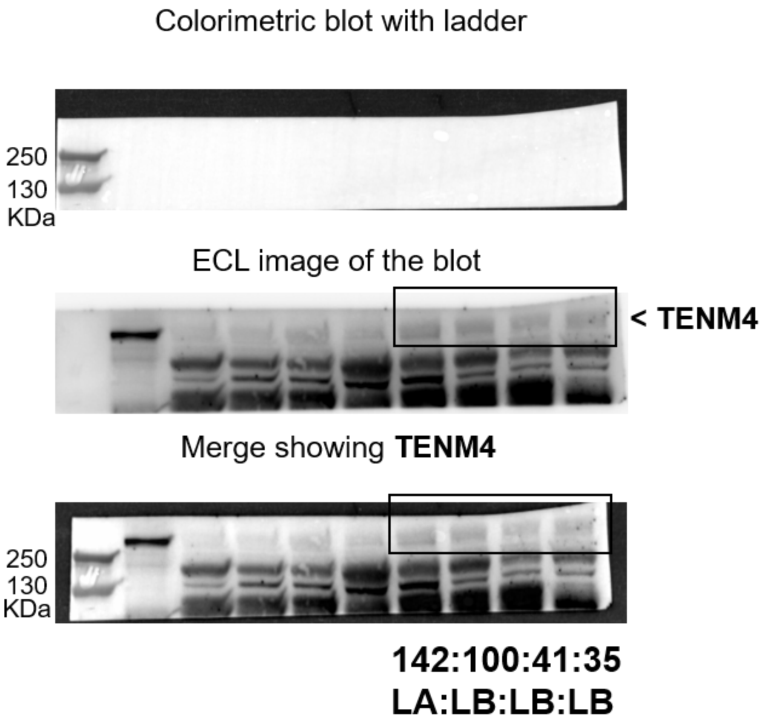

B) W. Blot Figure S3b, for **CD9**. Samples in black rectangles are shown.

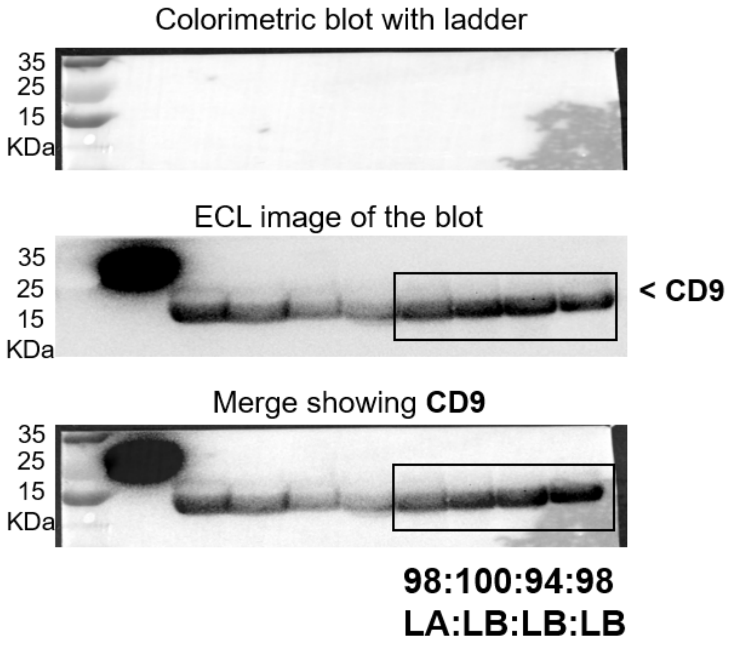

Supplement: Supplementary file 1 [file cancers-13-00894-s001.zip › Figure S5. original WB figures.pdf]
