# Supplementary material for: Identification of TENM4 as a Novel Cancer Stem Cell-Associated Molecule and Potential Target in Triple Negative Breast Cancer
Source: Cancers (Basel). 2021 Feb 20;13(4):894. doi: 10.3390/cancers13040894 (PMC7924390; doi:10.3390/cancers13040894)
Supplement: Supplementary file 1 [file cancers-13-00894-s001.zip › New Microsoft Word Document.docx]

Identification of TENM4 as a Novel Cancer Stem Cell-Associated Molecule and Potential Target in Triple Negative Breast Cancer

Roberto Ruiu, Giuseppina Barutello, Maddalena Arigoni, Federica Riccardo, Laura Conti, Giulia Peppino, Laura Annaratone, Caterina Marchiò, Giulio Mengozzi, Raffaele Adolfo Calogero, Federica Cavallo and Elena Quaglino


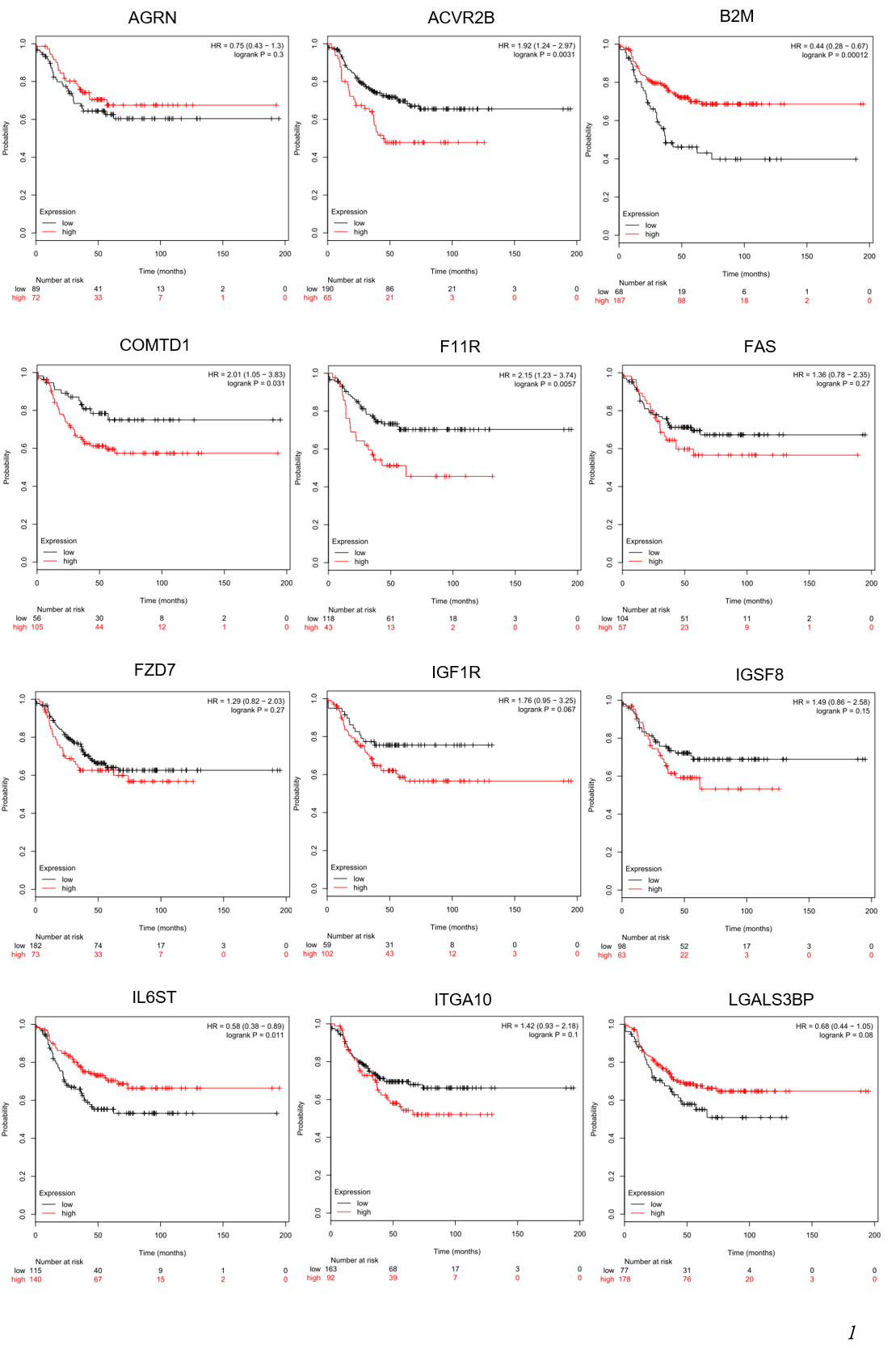


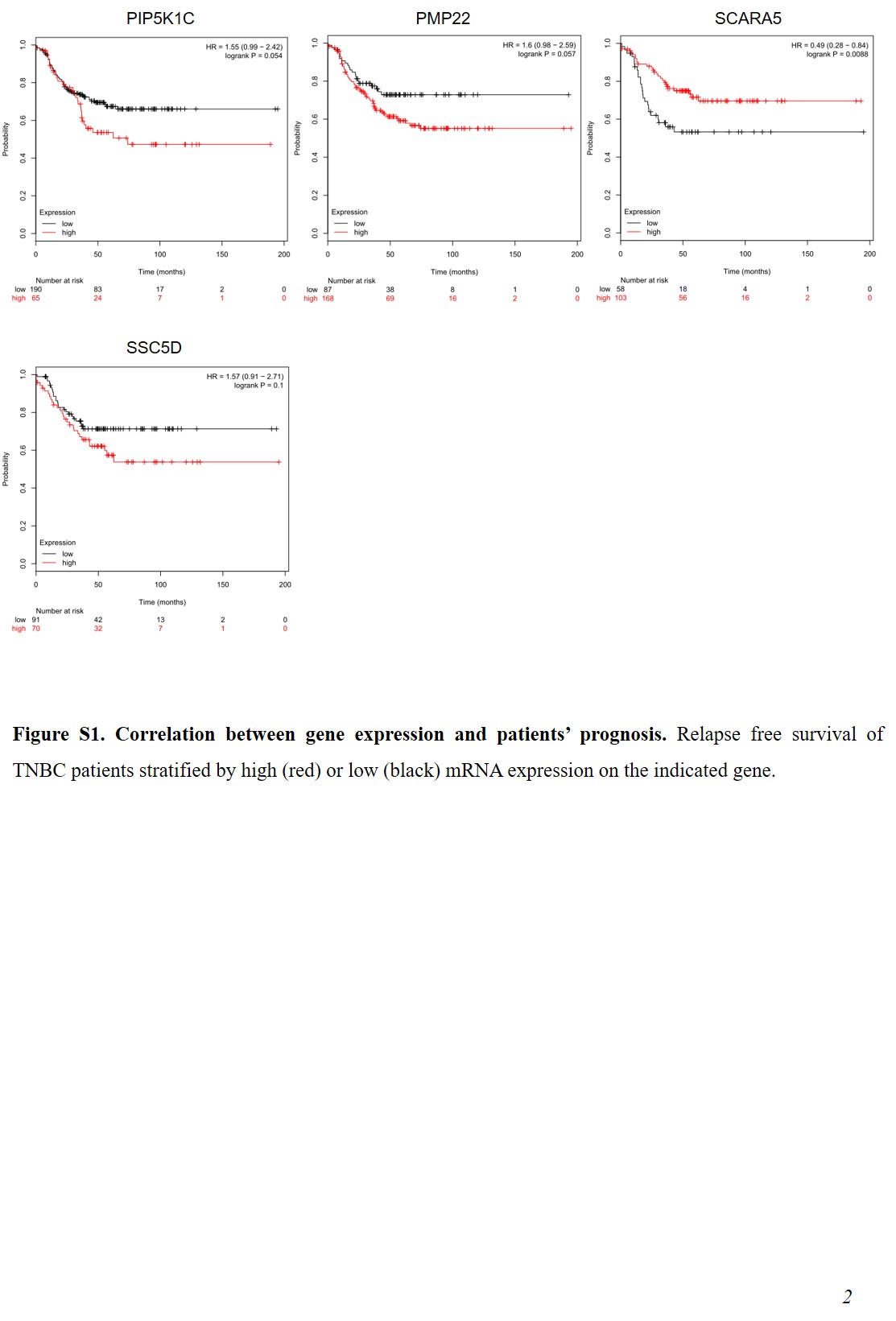


**Figure S1.** Correlation between gene expression and patients’ prognosis. Relapse free survival of TNBC patients stratified by high (red) or low (black) mRNA expression on the indicated gene.


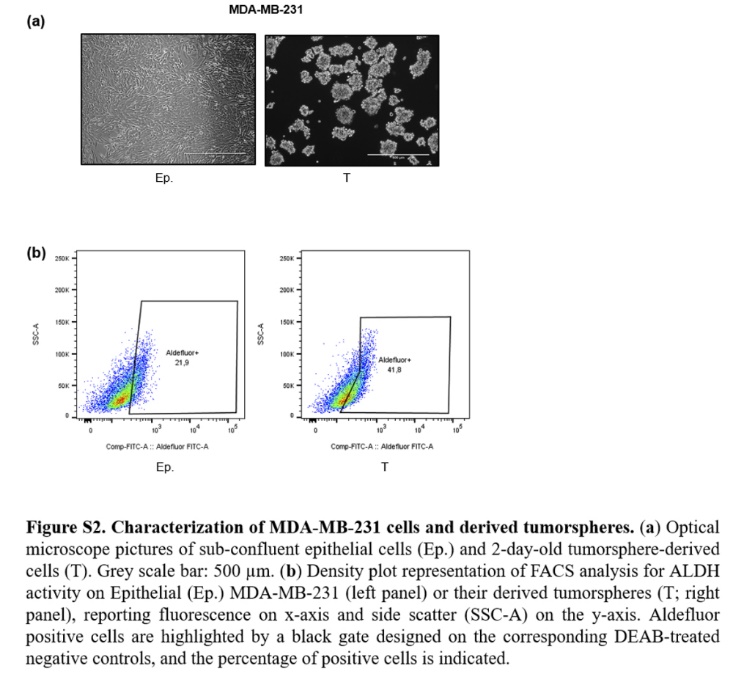


**Figure S2.** Characterization of MDA-MB-231 cells and derived tumorspheres. (**a**) Optical microscope pictures of sub-confluent epithelial cells (Ep.) and 2-day-old tumorsphere-derived cells (T). Grey scale bar: 500 μm. (**b**) Density plot representation of FACS analysis for ALDH activity on Epitelial (Ep) MDA-MB-231 (left panel) or their derived tumorspheres treated with control siRNA (T mock; central panel) or with siRNA for TENM4 (T siRNA; right panel), reporting fluorescence on x-axis and side scatter (SSC-A) on the y-axis. Aldefluor positive cells are highlighted by a black gate designed on the corresponding DEAB-treated negative controls, and the percentage of positive cells is indicated.


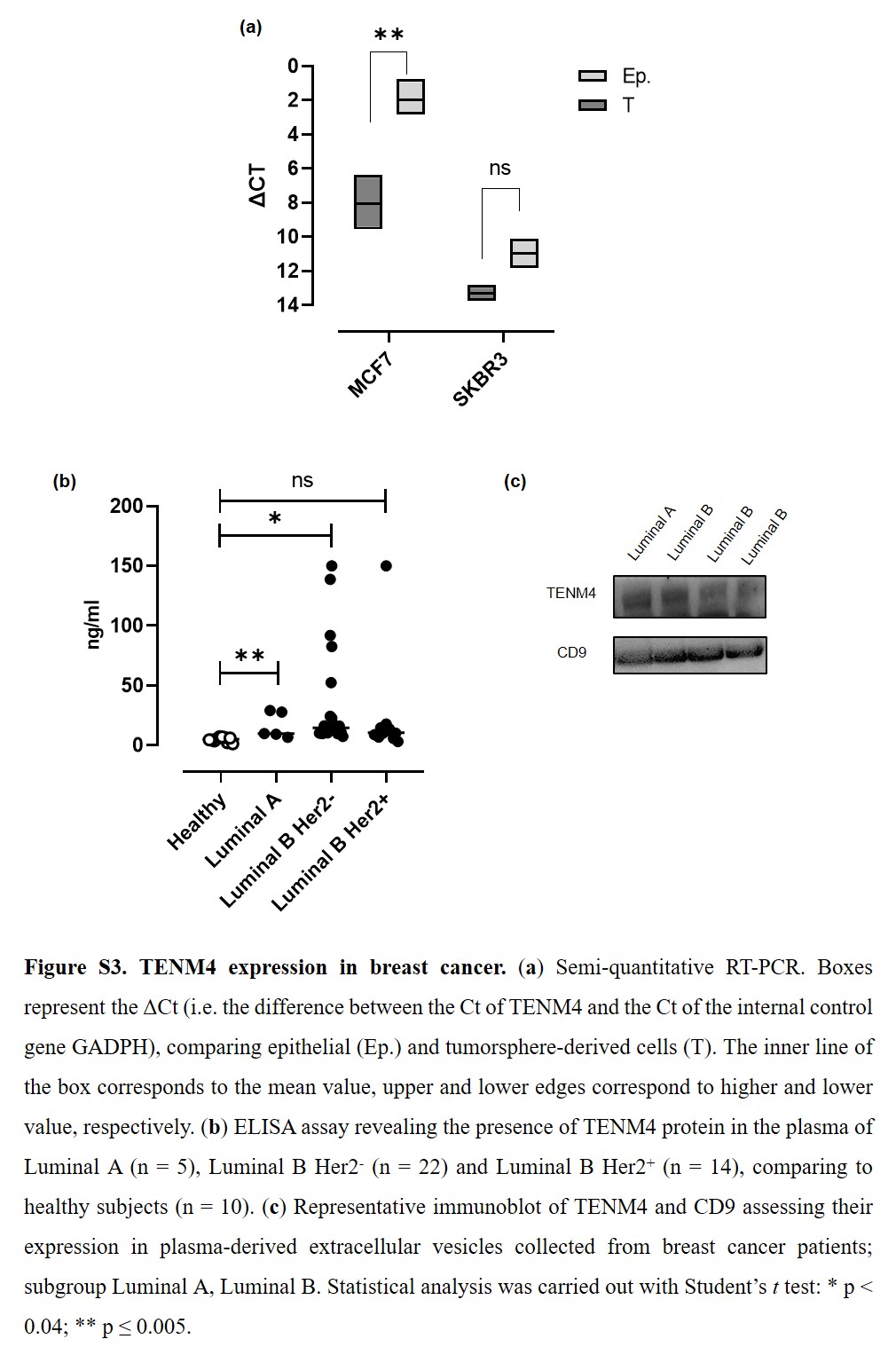


**Figure S3.** TENM4 expression in breast cancer. (**a**) Semi-quantitative RT-PCR. Boxes represent. the ΔCt (i.e. the difference between the Ct of TENM4 and the Ct of the internal control gene GADPH), comparing epithelial (Ep.) and tumorsphere-derived cells (T). The inner line of the box corresponds to the mean value, upper and lower edges correspond to higher and lower value, respectively. (**b**) Representative immunoblot of TENM4 and CD9 assessing their expression in plasma-derived extracellular vesicles collected from breast cancer patients; subgroup Luminal A, Luminal B. (**c**) ELISA assay revealing the presence of TENM4 protein in the plasma of Luminal A (*n* = 5), Luminal B Her2- (*n* = 22) and Luminal B Her2+ (*n* = 14), comparing to healthy subjects (*n* = 10). Statistical analysis was carried out with Student’s t test: * *p* < 0.04; ** *p* ≤ 0.005.


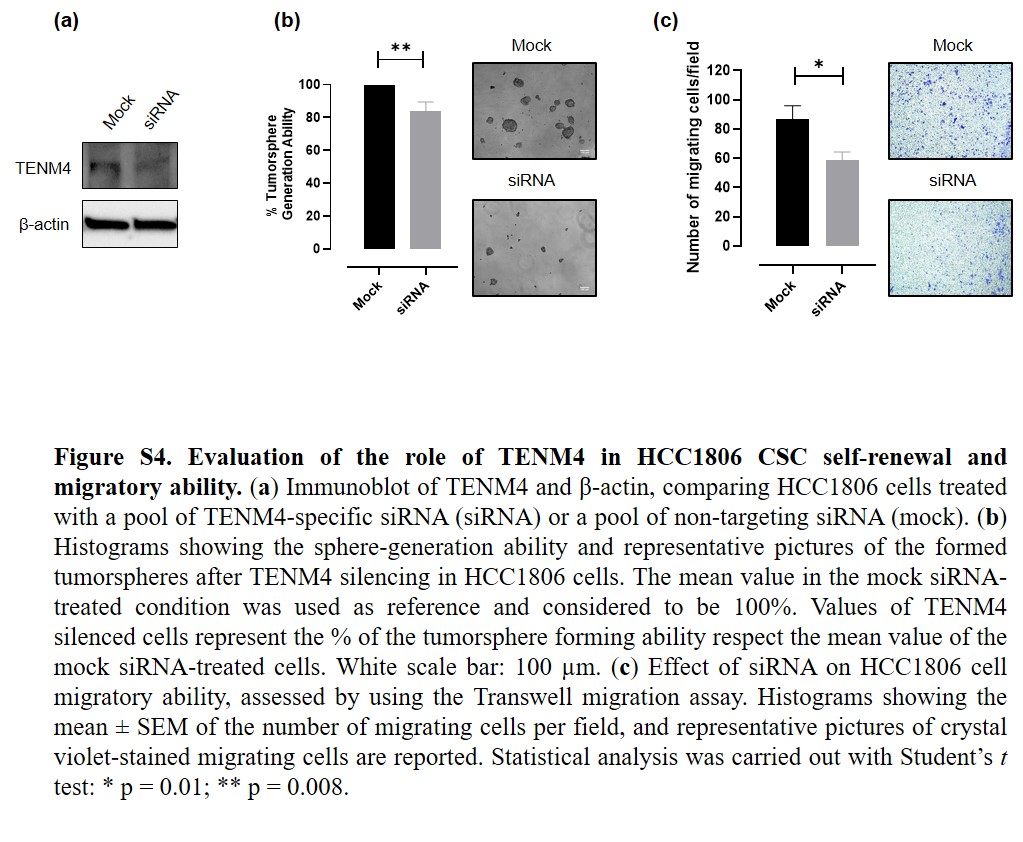


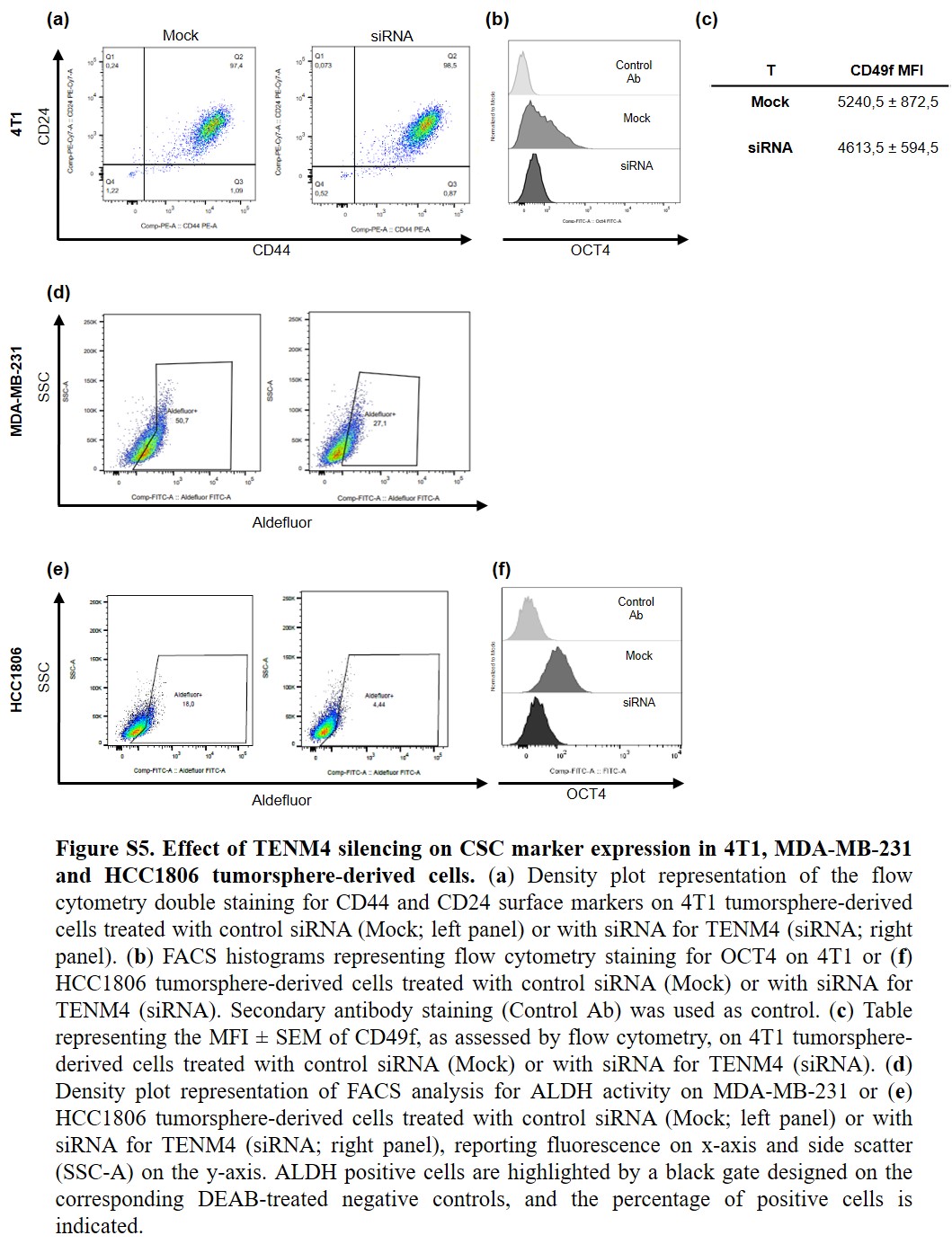


**Table S1.** Up-regulated genes in tumorspheres

| **GO Biological Function** | **Gene Symbols** |
| --- | --- |
| negative regulation of neuron death  (GO:1901215) | NR4A2, NRBP2, LGMN, CCL2, BDNF, IKBKG, HIPK2 |
| regulation of neuron death (GO:1901214) | NR4A2, CLU, NRBP2, LGMN, CCL2, BDNF, IKBKG,  AGRN, HIPK2 |
| regulation of neuron apoptotic process  (GO:0043523) | NR4A2, NRBP2, LGMN, CCL2, BDNF, AGRN, HIPK2 |
| response to bacterium (GO:0009617) | SSC5D, NOS2, B2M, BCL3, PTGS2, CCL2, ASS1, FUCA2,  SLPI, HIST1H2BK, MALT1 |
| response to biotic stimulus (GO:0009607) | SSC5D, NOS2, B2M, CLU, BCL3, PTGS2, IFI44, CCL2, ASS1, IKBKG, FUCA2, SLPI, HIST1H2BK, TXNIP,  MALT1 |
| regulation of cytokine production  (GO:0001817) | SSC5D, B2M, CLU, BCL3, PTGS2, F11R, IL6ST, CCL2,  IKBKG, BCL6, MALT1 |
| response to other organism (GO:0051707) | SSC5D, NOS2, B2M, CLU, BCL3, PTGS2, IFI44, CCL2,  ASS1, IKBKG, FUCA2, SLPI, HIST1H2BK, MALT1 |
| response to external biotic stimulus  (GO:0043207) | SSC5D, NOS2, B2M, CLU, BCL3, PTGS2, IFI44, CCL2,  ASS1, IKBKG, FUCA2, SLPI, HIST1H2BK, MALT1 |
| negative regulation of apoptotic process (GO:0043066) | NR4A2, CLU, BCL3, IGF1R, IL6ST, NRBP2, LGMN,  DUSP1, CCL2, BDNF, FAS, CYR61, BCL6, NR4A1, HIPK2, MALT1 |
| negative regulation of programmed cell death (GO:0043069) | NR4A2, CLU, BCL3, IGF1R, IL6ST, NRBP2, LGMN, DUSP1, CCL2, BDNF, FAS, CYR61, BCL6, NR4A1,  HIPK2, MALT1 |
| negative regulation of cell death (GO:0060548) | NR4A2, CLU, BCL3, IGF1R, IL6ST, NRBP2, LGMN, DUSP1, CCL2, BDNF, FAS, IKBKG, CYR61, BCL6,  NR4A1, HIPK2, MALT1 |
| enzyme linked receptor protein signaling pathway (GO:0007167) | MVP, ARID5B, TRPS1, ACVR2B, IGF1R, F11R, IL6ST,  CCL2, DNM1, BDNF, DUSP4, NR4A1, CSF1, HIPK2, TXNIP |
| innate immune response (GO:0045087) | SSC5D, NOS2, B2M, CLU, LGMN, CCL2, ASS1, IKBKG, DUSP4, HIST1H2BK, NR4A1, CSF1, TXNIP, MALT1,  MASP1 |
| defense response (GO:0006952) | SSC5D, NOS2, B2M, CLU, BCL3, PTGS2, F11R, LGMN, CCL2, ASS1, BDNF, IKBKG, BCL6, SLPI, DUSP4, HIST1H2BK, NR4A1, LGALS3BP, CSF1, TXNIP, MALT1,  MASP1 |
| regulation of apoptotic process (GO:0042981) | NR4A2, CLU, TRPS1, BCL3, PTGS2, IGF1R, IL6ST, NRBP2, LGMN, DUSP1, CCL2, BDNF, FAS, CYR61,  BCL6, AGRN, NR4A1, HIPK2, TXNIP, MALT1 |
| regulation of programmed cell death (GO:0043067) | NR4A2, CLU, TRPS1, BCL3, PTGS2, IGF1R, IL6ST,  NRBP2, LGMN, DUSP1, CCL2, BDNF, FAS, CYR61, BCL6, AGRN, NR4A1, HIPK2, TXNIP, MALT1 |
| immune response (GO:0006955) | SSC5D, NOS2, B2M, CLU, BCL3, IGF1R, LGMN, CCL2, ASS1, FAS, IKBKG, BCL6, SLPI, DUSP4, HIST1H2BK,  NR4A1, CSF1, TXNIP, MALT1, MASP1 |
| regulation of cell death (GO:0010941) | NR4A2, CLU, TRPS1, BCL3, PTGS2, IGF1R, IL6ST,  NRBP2, LGMN, DUSP1, CCL2, BDNF, FAS, IKBKG, CYR61, BCL6, AGRN, NR4A1, HIPK2, TXNIP, MALT1 |
| positive regulation of multicellular organismal process (GO:0051240) | NOS2, B2M, CLU, BCL3, PTGS2, ACVR2B, IL6ST, CCL2,  BDNF, IKBKG, CYR61, NBL1, BCL6, AGRN, CSF1, HIPK2, MALT1, **TENM4** |

| response to external stimulus (GO:0009605) | SSC5D, NR4A2, NOS2, B2M, CLU, BCL3, PTGS2, IGF1R, IFI44, ATG14, CCL2, DNM1, ASS1, BDNF, FAS, IKBKG, PIP5K1C, CYR61, FUCA2, AGRN, ITGA10, SLPI,  HIST1H2BK, NR4A1, TXNIP, MALT1 |
| --- | --- |
| positive regulation of biosynthetic process (GO:0009891) | NR4A2, NOS2, FOSL2, ARID5B, CLU, CEBPD, BCL3,  PTGS2, IGF1R, AHR, CCL2, ASS1, BDNF, CALCOCO1, IKBKG, CYR61, AGRN, FZD7, NR4A1, HIPK2, MALT1 |
| tissue development (GO:0009888) | BDH2, ARID5B, TRPS1, PTGS2, ACVR2B, IGF1R, F11R, DUSP1, IGSF8, BDNF, CYR61, DUSP4, FZD7, NR4A1,  CSF1, HIPK2, FNDC3A, TXNIP, **TENM4** |
| positive regulation of cellular biosynthetic process (GO:0031328) | NR4A2, NOS2, FOSL2, ARID5B, CLU, CEBPD, BCL3,  PTGS2, IGF1R, AHR, ASS1, BDNF, CALCOCO1, IKBKG, CYR61, AGRN, FZD7, NR4A1, HIPK2, MALT1 |
| neurogenesis (GO:0022008) | NR4A2, ZSWIM6, CLU, IGF1R, IL6ST, NRBP2, CCL2, DNM1, BDNF, PIP5K1C, NBL1, BCL6, AGRN, ITGA10,  FZD7, CSF1, GAS7, HIPK2, **TENM4** |
| positive regulation of nitrogen compound metabolic process (GO:0051173) | NR4A2, NOS2, FOSL2, ARID5B, CLU, CEBPD, BCL3, PTGS2, IGF1R, AHR, ASS1, BDNF, CALCOCO1, IKBKG,  CYR61, AGRN, FZD7, NR4A1, HIPK2, MALT1 |
| regulation of multicellular organismal process (GO:0051239) | SSC5D, NR4A2, NOS2, B2M, ZSWIM6, CLU, TRPS1, BCL3, PTGS2, ACVR2B, F11R, IL6ST, LGMN, CCL2, BDNF, FAS, IKBKG, CYR61, NBL1, BCL6, **SCARA5**,  AGRN, FZD7, CSF1, HIPK2, MALT1, **TENM4** |
| anatomical structure morphogenesis (GO:0009653) | NR4A2, ZSWIM6, CLU, ARID5B, PMP22, BCL3, PTGS2, ACVR2B, IGF1R, DUSP1, CCL2, DNM1, BDNF, PIP5K1C, CYR61, NBL1, BCL6, AGRN, ITGA10, DUSP4,  FZD7, NR4A1, CSF1, GAS7, HIPK2, **TENM4** |
| immune system process (GO:0002376) | SSC5D, NOS2, B2M, CLU, BCL3, IGF1R, LGMN, CCL2, ASS1, FAS, IKBKG, PIPK1C, BCL6, SLPI, DUSP4, FZD7, HIST1H2BK, NR4A1, CSF1, HIPK2, TXNIP, MALT1,  MASP1 |
| positive regulation of response to stimulus (GO:0048584) | CREBRF, B2M, CLU, TRPS1, PTGS2, ACVR2B, IGF1R, IL6ST, LGMN, ATG14, CCL2, BDNF, FAS, IKBKG,  CYR61, DUSP4, FZD7, CSF1, HIPK2, MALT1, MASP1 |
| cell surface receptor signaling pathway (GO:0007166) | MVP, B2M, ARID5B, TRPS1, ACVR2B, IGF1R, F11R, IL6ST, CCL2, DNM1, BDNF, FAS, CALCOCO1, IKBKG, ITGA10, DUSP4, FZD7, NR4A1, CSF1, HIPK2, TXNIP,  MALT1 |
| regulation of molecular function (GO:0065009) | MVP, NR4A2, NOS2, B2M, CLU, ARID5B, BCL3, ACVR2B, IGF1R, F11R, DUSP1, ATG14, CCL2, BDNF, FAS, IKBKG, CYR61, BCL6, AGRN, SLPI, DUSP4,  NR4A1, CSF1, HIPK2, KLHL24, TXNIP, MALT1, |
| regulation of protein metabolic process (GO:0051246) | MVP, NOS2, CREBRF, CLU, TRPS1, BCL3, ACVR2B, IGF1R, IL6ST, DUSP1, ATG14, CCL2, BDNF, FAS, IKBKG, CYR61, BCL6, SLPI, DUSP4, FZD7, NR4A1,  CSF1, HIPK2, MALT1, MASP1 |
| cell differentiation (GO:0030154) | BDH2, NR4A2, B2M, ZSWIM6, CLU, ARID5B, TRPS1, BCL3, PTGS2, ACVR2B, IGF1R, F11R, IL6ST, NRBP2, CCL2, DNM1, BDNF, FAS, PIP5K1C, CYR61, NBL1,  BCL6, AGRN, ITGA10, FZD7, NR4A1, CSF1, GAS7 |
| regulation of cellular protein metabolic process (GO:0032268) | MVP, CLU, TRPS1, BCL3, ACVR2B, IGF1R, IL6ST, DUSP1, ATG14, CCL2, BDNF, FAS, IKBKG, CYR61, BCL6, SLPI, DUSP4, FZD7, NR4A1, CSF1, HIPK2,  MALT1, MASP1 |

| cellular developmental process (GO:0048869) | BDH2, NR4A2, B2M, ZSWIM6, CLU, ARID5B, PMP22, TRPS1, BCL3, PTGS2, ACVR2B, IGF1R, F11R, IL6ST, NRBP2, CCL2, DNM1, BDNF, FAS, PIP5K1C, CYR61,  NBL1, BCL6, AGRN, ITGA10, FZD7, NR4A1, CSF1 |
| --- | --- |
| regulation of signal transduction (GO:0009966) | CREBRF, CLU, TRPS1, BCL3, PTGS2, ACVR2B, IGF1R, IL6ST, LGMN, DUSP1, CCL2, BDNF, FAS, IKBKG, CYR61, CLU, NBL1, BCL6, AGRN, DUSP4, FZD7, CSF1,  HIPK2, KLHL24, MALT1 |
| response to stress (GO:0006950) | SSC5D, NR4A2, NOS2, CREBRF, B2M, CLU, BCL3, PTGS2, F11R, LGMN, DUSP1, ATG14, CCL2, ASS1, BDNF, FAS, IKBKG, PIP5K1C, CYR61, PLA2G4A, BCL6, **SCARA5**, ITGA10, SLPI, DUSP4, FZD7, HIST1H2BK,  NR4A1, LGALS3BP |
| positive regulation of cellular metabolic process (GO:0031325) | NR4A2, NOS2, FOSL2, CLU, ARID5B, CEBPD, BCL3, PTGS2, ACVR2B, IGF1R, AHR, IL6ST, ATG14, CCL2, ASS1, BDNF, FAS, CALCOCO1, IKBKG, CYR61, BCL6,  AGRN, FZD7, NR4A1, CSF1, HIPK2, MALT1 |
| positive regulation of macromolecule metabolic process (GO:0010604) | NR4A2, CREBRF, FOSL2, CLU, ARID5B, CREBPD, BCL3, ACVR2B, IGF1R, AHR, IL6ST, ATG14, CCL2, BDNF, FAS, CALCOCO1, IKBKG, CYR61, BCL6, AGRN,  FZD7, NR4A1, CSF1, HIPK2, MALT1 |
| regulation of signaling (GO:0023051) | MVP, NOS2, CREBRF, CLU, TRPS1, BCL3, PTGS2, ACVR2B, IGF1R, IL6ST, LGMN, DUSP1, CCL2, BDNF,  FAS, IKBKG, CYR61, NBL1, BCL6, AGRN, DUSP4, FZD7, CSF1, HIPK2, KLHL24, MALT1 |
| regulation of cell communication (GO:0010646) | NOS2, CREBRF, CLU, TRPS1, BCL3, PTGS2, ACVR2B, IGF1R, IL6ST, NRBP2, LGMN, DUSP1, ATG14, CCL2,  BDNF, FAS, IKBKG, CYR61, NBL1, BCL6, AGRN, DUSP4, FZD7, CSF1, HIPK2, KLHL24, MALT1 |
| system development (GO:0048731) | NR4A2, B2M, ZSWIM6, CLU, ARID5B, PMP22, TRPS1, BCL3, PTGS2, ACVR2B, IGF1R, AHR, IL6ST, NRBP2, CCL2, IGSF8, DNM1, ASS1, BDNF, FAS, PIP5K1C, CYR61, NBL1, BCL6, AGRN, ITGA10, FZD7, NR4A1,  CSF1 |
| negative regulation of cellular process (GO:0048523) | MVP, SSC5D, NR4A2, CREBRF, CLU, ARID5B, TRPS1, BCL3, PTGS2, ACVR2B, IGF1R, AHR, IL6ST, NRBP2, LGMN, DUSP1, ATG14, CCL2, ASS1, BDNF, FAS, IKBKG, CYR61, NBL1, BCL6, SLPI, DUSP4, FZD7,  NR4A1 |
| regulation of response to stimulus (GO:0048583) | CREBRF, B2M, CLU, TRPS1, BCL3, PTGS2, ACVR2B, IGF1R, IL6ST, NRBP2, LGMN, DUSP1, ATG14, CCL2, BDNF, FAS, IKBKG, CYR61, NBL1, BCL6, **SCARA5**,  AGRN, DUSP4, FZD7, NR4A1, CSF1, HIPK2, KLHL24 |
| multicellular organismal development (GO:0007275) | SSC5D, NR4A2, B2M, ZSWIM6, CLU, ARID5B, PMP22, TRPS1, BCL3, PTGS2, ACVR2B, IGF1R, AHR, IL6ST,  NRBP2, DUSP1, CCL2, IGSF8, DNM1, ASS1, BDNF, FAS, PIP5K1C, CYR61, NBL1, BCL6, AGRN, ITGA10, DUSP4 |
| anatomical structure development (GO:0048856) | BDH2, NR4A2, B2M, ZSWIM6, CLU, ARID5B, PMP22, TRPS1, BCL3, PTGS2, ACVR2B, IGF1R, AHR, F11R, IL6ST, NRBP2, DUSP1, CCL2, IGSF8, DNM1, ASS1,  BDNF, FAS, PIP5K1C, CYR61, NBL1, BCL6, AGRN, ITGA10 |
| negative regulation of biological process  (GO:0048519) | MVP, SSC5D, NR4A2, NOS2, CREBRF, CLU, ARID5B,  TRPS1, BCL3, PTGS2, ACVR2B, IGF1R, AHR, F11R, |

|  | IL6ST, NRBP2, LGMN, DUSP1, ATG14, CCL2, ASS1,  BDNF, FAS, IKBKG, CYR61, NBL1, BCL6, SLPI, DUSP4 |
| --- | --- |
| positive regulation of metabolic process (GO:0009893) | NR4A2, NOS2, CREBRF, FOSL2, CLU, ARID5B, CEBPD, BCL3, PTGS2, ACVR2B, IGF1R, AHR, F11R, IL6ST, ATG14, CCL2, ASS1, BDNF, FAS, CALCOCO1, IKBKG,  CYR61, BCL6, AGRN, FZD7, NR4A1, CSF1, HIPK2 |
| developmental process (GO:0032502) | BDH2, SSC5D, NR4A2, B2M, ZSWIM6, CLU, ARID5B, PMP22, TRPS1, PNPLA7, BCL3, PTGS2, ACVR2B, IGF1R, AHR, F11R, IL6ST, NRBP2, DUSP1, CCL2, IGSF8,  DNM1, ASS1, BDNF, FAS, PIP5K1C, CYR61, NBL1, BCL6 |
| single-organism developmental process (GO:0044767) | BDH2, SSC5D, NR4A2, B2M, ZSWIM6, CLU, ARID5B, PMP22, TRPS1, BCL3, PTGS2, ACVR2B, IGF1R, AHR, F11R, IL6ST, NRBP2, DUSP1, CCL2, IGSF8, DNM1,  ASS1, BDNF, FAS, PIP5K1C, CYR61, NBL1, BCL6, AGRN |
| positive regulation of cellular process (GO:0048522) | NR4A2, NOS2, CREBRF, B2M, FOSL2, CLU, ARID5B, TRPS1, CEBPD, BCL3, PTGS2, ACVR2B, IGF1R, AHR, IL6ST, ATG14, CCL2, ASS1, BDNF, FAS, CALCOCO1,  IKBKG, CYR61, NBL1, BCL6, AGRN, FZD7, NR4A1 |
| signal transduction (GO:0007165) | MVP,NR4A2, NOS2, B2M, CLU, ARID5B, TRPS1, BCL3, ACVR2B, IGF1R, ARL5B, AHR, F11R, IL6ST, LGMN, DUSP1, PLEKHM3, CCL2, DNM1, BDNF, FAS,  CALCOCO1, IKBKG, CYR61, BCL6, AGRN, ITGA10, DUSP4, FZD7 |
| positive regulation of biological process (GO:0048518) | NR4A2, NOS2, CREBRF, B2M, FOSL2, CLU, ARID5B, TRPS1, CEBPD, BCL3, PTGS2, ACVR2B, IGF1R, AHR, F11R, IL6ST, LGMN, DUSP1, ATG14, CCL2, ASS1, BDNF, FAS, CALCOCO1, IKBKG, CYR61, NBL1, BCL6,  AGRN |
| cell communication (GO:0007154) | MVP,NR4A2, NOS2, B2M, CLU, ARID5B, PMP22, TRPS1, BCL3, ACVR2B, IGF1R, ARL5B, AHR, F11R, IL6ST, LGMN, DUSP1, PLEKHM3, ATG14, CCL2, DNM1, BDNF, FAS, CALCOCO1, IKBKG, CYR61, BCL6,  AGRN |
| single organism signaling (GO:0044700) | MVP,NR4A2, NOS2, B2M, CLU, ARID5B, PMP22, TRPS1, BCL3, ACVR2B, IGF1R, ARL5B, AHR, F11R, IL6ST, LGMN, DUSP1, PLEKHM3, CCL2, DNM1, BDNF, FAS, CALCOCO1, IKBKG, CYR61, BCL6, AGRN,  ITGA10 |
| signaling (GO:0023052) | MVP,NR4A2, NOS2, B2M, CLU, ARID5B, PMP22, TRPS1, BCL3, ACVR2B, IGF1R, ARL5B, AHR, F11R, IL6ST, LGMN, DUSP1, PLEKHM3, CCL2, DNM1, BDNF,  FAS, CALCOCO1, IKBKG, CYR61, BCL6, AGRN, ITGA10 |
| cellular response to stimulus (GO:0051716) | MVP,NR4A2, NOS2, CREBRF, B2M, CLU, ARID5B, TRPS1, BCL3, PTGS2, ACVR2B, IGF1R, ARL5B, AHR, F11R, IL6ST, LGMN, DUSP1, PLEKHM3, ATG14, CCL2, DNM1, BDNF, FAS, CALCOCO1, IKBKG, PIP5K1C,  CYR61 |
| single-multicellular organism process (GO:0044707) | SSC5D, NR4A2, NOS2, B2M, ZSWIM6, CLU, ARID5B, PMP22, TRPS1, BCL3, PTGS2, ACVR2B, IGF1R, AHR, IL6ST, NRBP2, LGMN, DUSP1, CCL2, IGSF8, DNM1,  ASS1, BDNF, FAS, PIP5K1C, CYR61, NBL1, PLA2G4A |

| multicellular organismal process (GO:0032501) | SSC5D, NR4A2, NOS2, CREBRF, B2M, ZSWIM6, CLU, ARID5B, PMP22, TRPS1, BCL3, PTGS2, ACVR2B, IGF1R, AHR, F11R, IL6ST, NRBP2, LGMN, DUSP1,  CCL2, IGSF8, DNM1, ASS1, BDNF, FAS, PIP5K1C, CYR61, NBL1 |
| --- | --- |
| response to stimulus (GO:0050896) | MVP, SSC5D, NR4A2, NOS2, CREBRF, B2M, CLU, ARID5B, TRPS1, BCL3, PTGS2, ACVR2B, IGF1R, ARL5B, AHR, F11R, IL6ST, LGMN, DUSP1, IFI44, PLEKHM3, ATG14, CCL2, DNM1, ASS1, BDNF, FAS,  CALCOCO1, IKBKG, PIP5K1C |
| Unclassified (UNCLASSIFIED) | RNF122, MFAP3L, LRRN4CL, TCP11L2 |

**Table S2.** Down-regulated genes in tumorspheres.

| **GO Biological Function** | **Gene Symbols** |
| --- | --- |
| isopentenyl diphosphate biosynthetic process, mevalonate pathway (GO:0019287) | MVK, PMVK, MVD |
| isopentenyl diphosphate biosynthetic  process (GO:0009240) | MVK, PMVK, MVD |
| isopentenyl diphosphate metabolic  process (GO:0046490) | MVK, PMVK, MVD |
| cholesterol biosynthetic process  (GO:0006695) | CYP51A1, MVK, PMVK, SQLE, MSMO1, DHCR7, MVD,  IDI1 |
| sterol biosynthetic process (GO:0016126) | CYP51A1, MVK, PMVK, SQLE, MSMO1, DHCR7, MVD,  IDI1 |
| isoprenoid biosynthetic process  (GO:0008299) | MVK, PMVK, MVD, IDI1 |
| cholesterol metabolic process  (GO:0008203) | CYP51A1, MVK, PMVK, SQLE, MSMO1, DHCR7,  PCSK9, MVD, IDI1 |
| sterol metabolic process (GO:0016125) | CYP51A1, MVK, PMVK, SQLE, MSMO1, DHCR7,  PCSK9, MVD, IDI1 |
| steroid biosynthetic process  (GO:0006694) | CYP51A1, MVK, PMVK, SQLE, MSMO1, DHCR7, MVD,  IDI1 |
| alcohol biosynthetic process  (GO:0046165) | CYP51A1, MVK, PMVK, SQLE, MSMO1, DHCR7, MVD,  IDI1 |
| mitotic prometaphase (GO:0000236) | CDC20, CCNB2, KIF2C, CENPM, ERCCL6, CENPU |
| organic hydroxy compound biosynthetic  process (GO:1901617) | CYP51A1, MVK, PMVK, SQLE, MSMO1, DHCR7, MVD,  IDI1 |
| steroid metabolic process (GO:0008202) | CYP51A1, MVK, PMVK, SQLE, MSMO1, DHCR7,  CYP24A1, PCSK9, MVD, IDI1 |
| anaphase (GO:0051322) | CDC20, KIF2C, ESPL1, CENPM, ERCC6L, CENPU |
| mitotic anaphase (GO:0000090) | CDC20, KIF2C, ESPL1, CENPM, ERCC6L, CENPU |
| mitotic M phase (GO:0000087) | CDC20, CCNB2, KIF2C, ESPL1, CENPM, ERCC6L,  KIF23, CENPU |
| M phase (GO:0000279) | CDC20, CCNB2, KIF2C, ESPL1, CENPM, ERCC6L,  KIF23, CENPU |
| alcohol metabolic process (GO:0006066) | CYP51A1, MVK, PMVK, SQLE, MSMO1, DBI, DHCR7,  CYP24A1, PCSK9, MVD, IDI1 |
| mitotic nuclear division (GO:0007067) | CDC20, CCNB2, KIF2C, MYBL2, CEP55, ESPL1,  ERCC6L, CDCA3, KIF23, FAM83D |
| mitotic cell cycle phase (GO:0098763) | CDC20, CCNB2, KIF2C, ESPL1, CENPM, ERCC6L,  KIF23, CENPU |

| cell cycle phase (GO:0022403) | CDC20, CCNB2, KIF2C, ESPL1, CENPM, ERCC6L,  KIF23, CENPU |
| --- | --- |
| biological phase (GO:0044848) | CDC20, CCNB2, KIF2C, ESPL1, CENPM, ERCC6L,  KIF23, CENPU |
| nuclear division (GO:0000280) | CDC20, CCNB2, KIF2C, MYBL2, CEP55, ESPL1,  ERCC6L, CDCA3, KIF23, FAM83D |
| organic hydroxy compound metabolic  process (GO:1901615) | CYP51A1, MVK, PMVK, SQLE, MSMO1, DHCR7,  CYP24A1, PCSK9, MVD, IDI1 |
| small molecule biosynthetic process  (GO:0044283) | CYP51A1, MVK, PMVK, SQLE, MSMO1, FADS2,  DHCR7, MVD, IDI1 |
| cell division (GO:0051301) | CDC20, CCNB2, KIF2C, CEP55, TUBA1C, ESPL1,  ERCC6L, CDCA3, KIF23, FAM83D |
| organelle fission (GO:0048285) | CDC20, CCNB2, KIF2C, MYBL2, CEP55, ESPL1,  ERCC6L, CDCA3, KIF23, FAM83D |
| lipid biosynthetic process (GO:0008610) | CYP51A1, MVK, PMVK, SQLE, MSMO1, FADS2,  DHCR7, MVD, IDI1 |
| mitotic cell cycle (GO:0000278) | CDC20, CCNB2, KIF2C, MYBL2, CEP55, ESPL1,  CENPM, ERCC6L, CDCA3, TTK, KIF23, FAM83D, CENPU |
| mitotic cell cycle process (GO:1903047) | CDC20, CCNB2, KIF2C, MYBL2, CEP55, ESPL1,  ERCC6L, CDCA3, TTK, KIF23, FAM83D |
| lipid metabolic process (GO:0006629) | CYP51A1, MVK, PMVK, SQLE, MSMO1, AACS, FADS2, DBI, DHCR7, ACAT2, CYP24A1, PCSK9, MVD, IDI1,  ARSJ |
| cell cycle (GO:0007049) | CDC20, CCNB2, KIF2C, MYBL2, CEP55, ESPL1,  CENPM, ERCC6L, CDCA3, TTK, MND1, KIF23, FAM83D, CENPU |
| cell cycle process (GO:0022402) | CDC20, CCNB2, KIF2C, MYBL2, CEP55, ESPL1,  ERCC6L, CDCA3, TTK, KIF23, FAM83D |
| small molecule metabolic process (GO:0044281) | CYP51A1, MVK, PMVK, SQLE, MSMO1, AACS, FADS2,  CDA, DBI, DHCR7, CYP24A1, PCSK9, MVD, TK1, IDI1, ARSJ |
| single-organism process (GO:0044699) | CDC20, CYP51A1, MVK, CCNB2, SGK2, KIF2C, PMBK, SQLE, DEPDC1B, MSMO1, MYBL2, AACS, CEP55, FADS2, CDA, MDM1, ITGA2, TUBA1C, HBEGF, RIPK3, ESPL1, CENPM, ERCC6L, CDCA3, TTK, DBI, DHCR7,  ACAT2, MND1, CYP24A1 |
| Unclassified (UNCLASSIFIED) | SAPCD2, CEP128 |
